# Supplementary material for: An in vivo and in vitro spatiotemporal profile of human midbrain development
Source: Nat Commun. 2026 Feb 3;17:1354. doi: 10.1038/s41467-025-67779-1 (PMC12877092; doi:10.1038/s41467-025-67779-1)

SUPPLEMENTARY INFORMATION

An in vivo and in vitro spatiotemporal profile of human midbrain development

Dimitri Budinger[1,#], Pau Puigdevall[2,3,#], George T. Hall[3,#], Charlotte Roth[1], Theodoros Xenakis[3], Elena Marrosu[1], Julie Jerber[4], Alessandro Di Domenico[1], Francesca Picco[1], Helena Kilpinen[2,5,6], Sergi Castellano[3], Manju A. Kurian[1,7,+], Serena Barral[1,*,+]

1. Developmental Neurosciences, Zayed Centre for Research, UCL Great Ormond Street Institute of Child Health, University College London, London, United Kingdom
2. Helsinki Institute of Life Science (HiLIFE), University of Helsinki, Helsinki, Finland
3. Genetics and Genomics Medicine, UCL Great Ormond Street Institute of Child Health, University College London, London, United Kingdom
4. Wellcome Sanger Institute, Wellcome Genome Campus, Hinxton, United Kingdom
5. Faculty of Biological and Environmental Sciences, University of Helsinki, Helsinki, Finland
6. Faculty of Medicine, University of Helsinki, Helsinki, Finland
7. Department of Neurology, Great Ormond Street Hospital, London, United Kingdom

# These authors contributed equally to the paper

+ These authors equally supervised the project

* Correspondence: Prof Manju A. Kurian (M.A.K.), manju.kurian@ucl.ac.uk or Dr Serena Barral (S.B), s.barral@ucl.ac.uk; Zayed Centre for Rare Diseases in Children, 20 Guilford Street, WC1N 1DZ, London, United Kingdom.

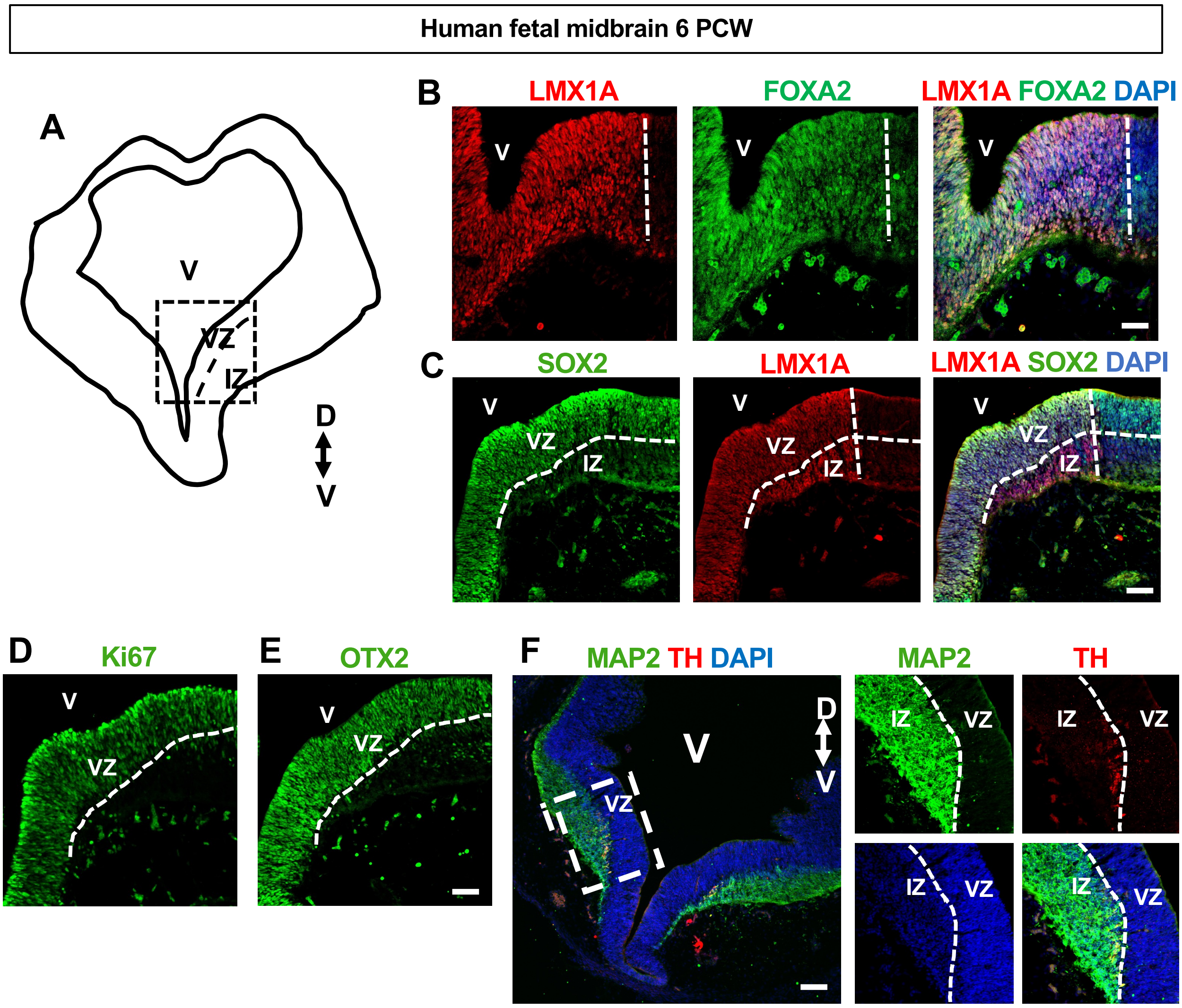

Human fetal midbrain 6 PCW
A
V
VZ
IZ
D
V
B
LMX1A
FOXA2
LMX1A FOXA2 DAPI
C
SOX2
LMX1A
LMX1A SOX2 DAPI
D
Ki67
E
OTX2
F
MAP2 TH DAPI
MAP2
TH

Supplementary Fig 1 Human ventral midbrain at early stage of development. A, Graphical representation of a coronal section of the human midbrain at 6 PCW. Created in BioRender. BUDINGER, D. (2025) https://BioRender.com/wn4tv4q. **B**, Immunofluorescence for LMX1A and FOXA2 in the midbrain ventral ventricular zone (VZ). **C**, Immunofluorescence staining for SOX2 and LMX1A in the ventral midbrain. **D-E**, Staining for Ki67 and OTX2 of the human midbrain at 6 PCW. **F**, Immunofluorescence analysis of a coronal section of the human ventral midbrain at 6 PCW for TH and MAP2. Nuclei are stained for DAPI. Lines demarcate the ventral midbrain region and the VZ to IZ boundary Scale bars = 100 μm (B, C, D and E); 200 μm (F). Ventricular zone (VZ), intermediate zone (IZ), ventricle (V).

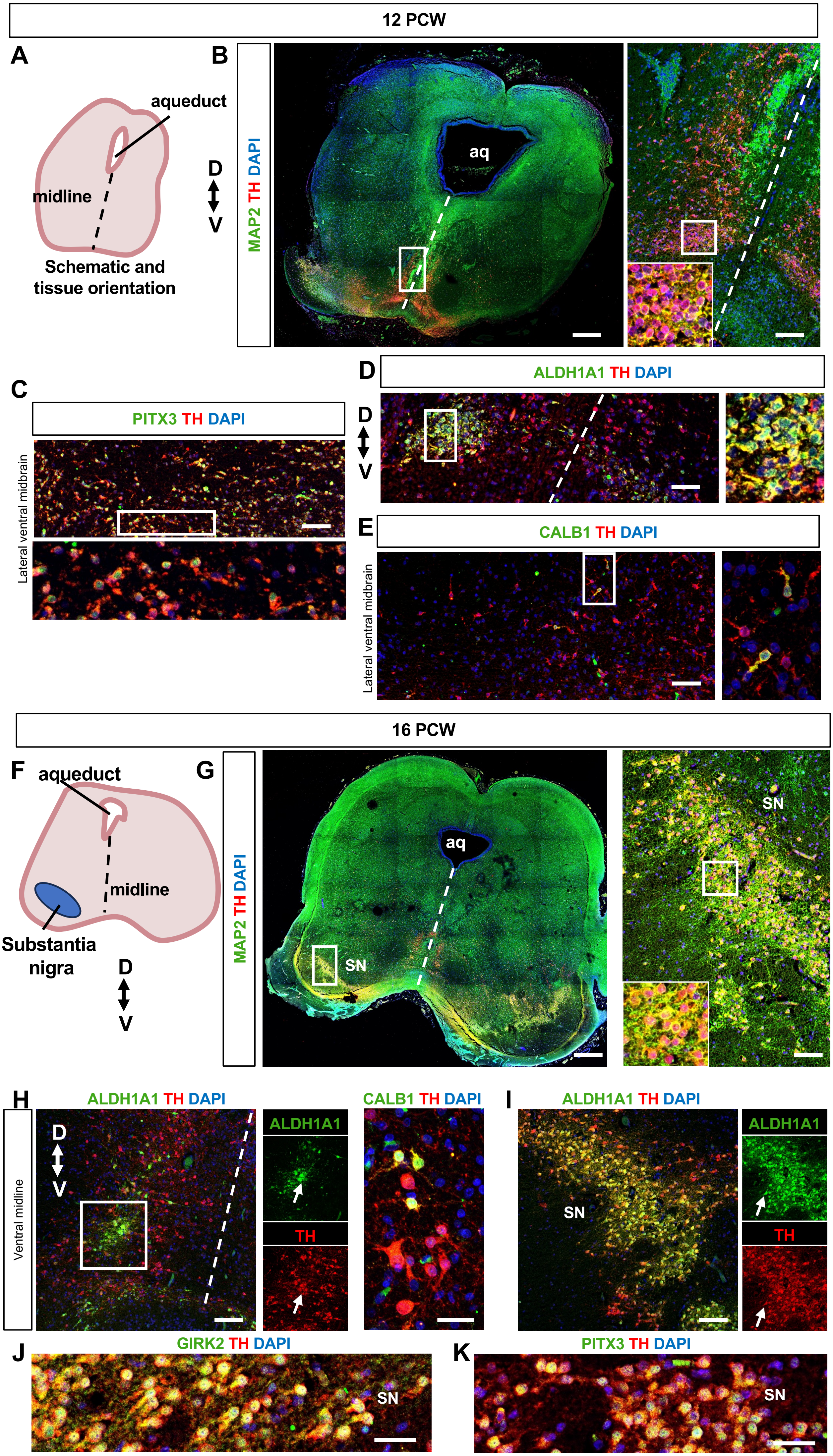

12 PCW
A
aqueduct
midline
Schematic and tissue orientation
B
D
V
MAP2 TH DAPI
aq
C
PITX3 TH DAPI
Lateral ventral midbrain
D
ALDH1A1 TH DAPI
E
CALB1 TH DAPI
Lateral ventral midbrain
16 PCW
F
aqueduct
midline
Substantia nigra
G
MAP2 TH DAPI
aq
SN
H
ALDH1A1 TH DAPI
Ventral midline
ALDH1A1
TH
CALB1 TH DAPI
I
ALDH1A1 TH DAPI
SN
J
GIRK2 TH DAPI
K
PITX3 TH DAPI

Supplementary Fig 2 Human midbrain maturation during the early second trimester of fetal development. A, Graphical representation of a coronal section of the human midbrain at 12 PCW. Created in BioRender. BUDINGER, D. (2025) https://BioRender.com/ynycjl1. **B**, Immunofluorescence analysis of human fetal sample at 12 PCW for MAP2 and TH. Scale bars = 300 µm (*left panel*) and 100 µm (*right panel*). **C**, Immunofluorescence representative image of the lateral ventral human midbrain at 12 PCW for PITX3 and TH. Scale bar = 100 µm. **D**, Representative images of immunofluorescence analysis for ALDH1A1 and TH at the human ventral midbrain ventral midline. Scale bar = 100 µm. **E**, Immunofluorescence for CALB1 and TH of the lateral ventral midbrain. Scale bar = 100 µm. **F**, Graphical representation of a coronal section of the human midbrain at 16 PCW. Created in BioRender. BUDINGER, D. (2025) https://BioRender.com/rgcf05m. **G**, Immunofluorescence analysis of human fetal sample at 16 PCW for MAP2 and TH. Scale bars = 400 µm (*left panel*) and 100 µm (*right panel*). **H**, Immunofluorescence analysis of the 16 PCW human midbrain in the ventral midbrain areas for ALDH1A1, CALB1 and TH. Scale bars = 100 µm (*left panel*) and 20 µm (*right panel*). **I**, Immunofluorescence analysis for ALDH1A1 and TH across the lateral ventral midbrain. Scale bar = 100 µm. **J-K**, Immunofluorescence analysis for GIRK2, PITX3 and TH in the lateral ventral midbrain. Scale bar = 30 µm. Nuclei are stained for DAPI. Lines demarcate ventral midline. Aqueduct (aq), dorsal (D), ventral (V), substantia nigra (SN).

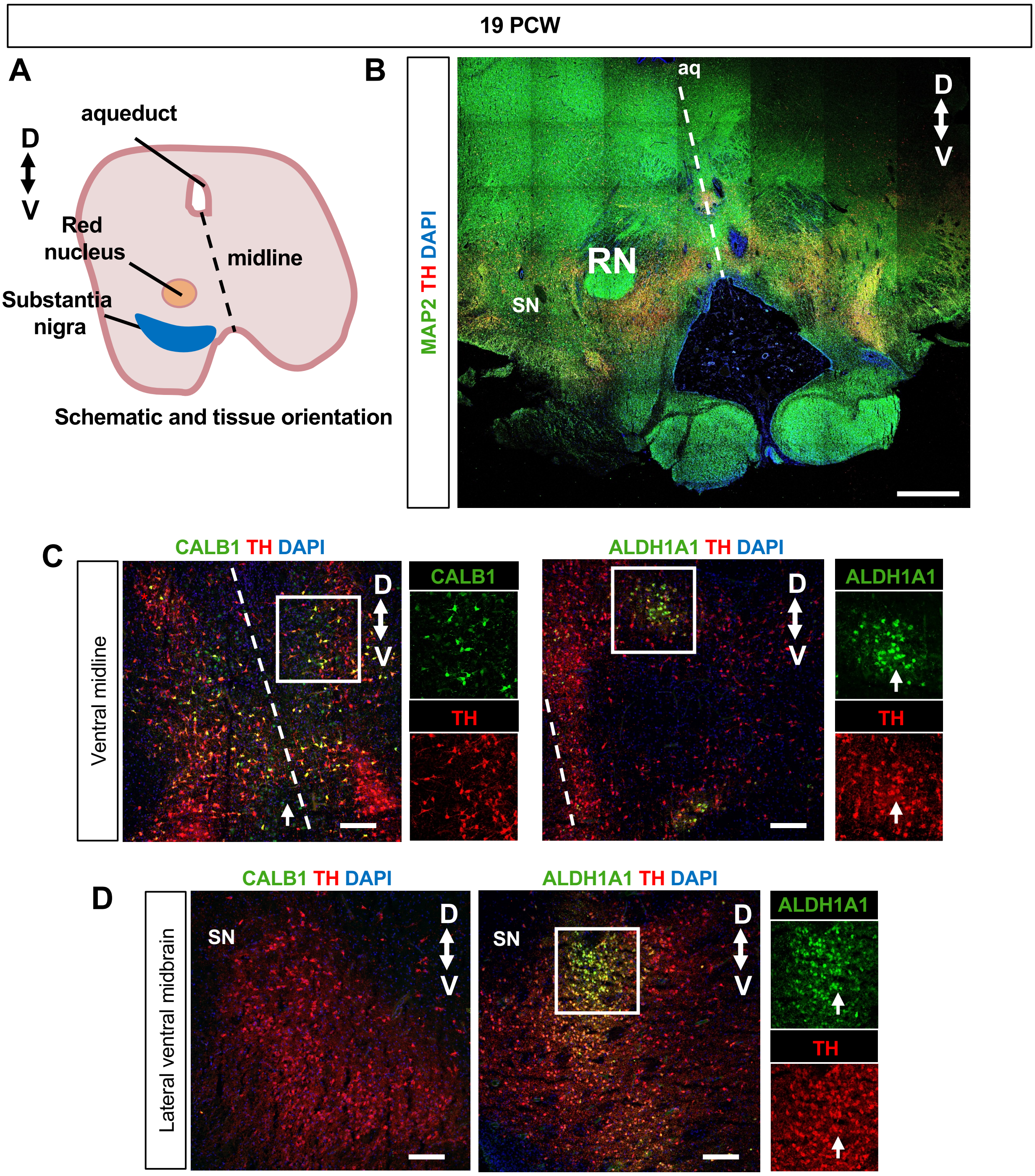
19 PCW
A
aqueduct
D
V
Red nucleus
midline
Substantia nigra
Schematic and tissue orientation
B
MAP2 TH DAPI
aq
D
V
RN
SN
C
Ventral midline
CALB1 TH DAPI
CALB1
TH
ALDH1A1 TH DAPI
ALDH1A1
TH
D
V
D
Lateral ventral midbrain
CALB1 TH DAPI
SN
ALDH1A1 TH DAPI
SN
ALDH1A1
TH

Supplementary Fig 3 Ventral midbrain dopaminergic neurons subtypes distribution at 19 PCW. A, Graphical representation of a coronal section of the human midbrain at 19 PCW. Created in BioRender. BUDINGER, D. (2025) https://BioRender.com/yujjdei. **B**, Immunofluorescence analysis of human fetal sample at 19 PCW for MAP2 and TH. Scale bar = 300 μm. **C-D**, Representative images of immunostaining for CALB1, ALDH1A1 and TH along the ventral midline and the lateral ventral midbrain of the human midbrain at 19 PCW, respectively. Scale bars = 100 μm. Nuclei are stained for DAPI. Lines demarcate ventral midline. Aqueduct (aq), dorsal (D), ventral (V), substantia nigra (SN), red nucleus (RN).

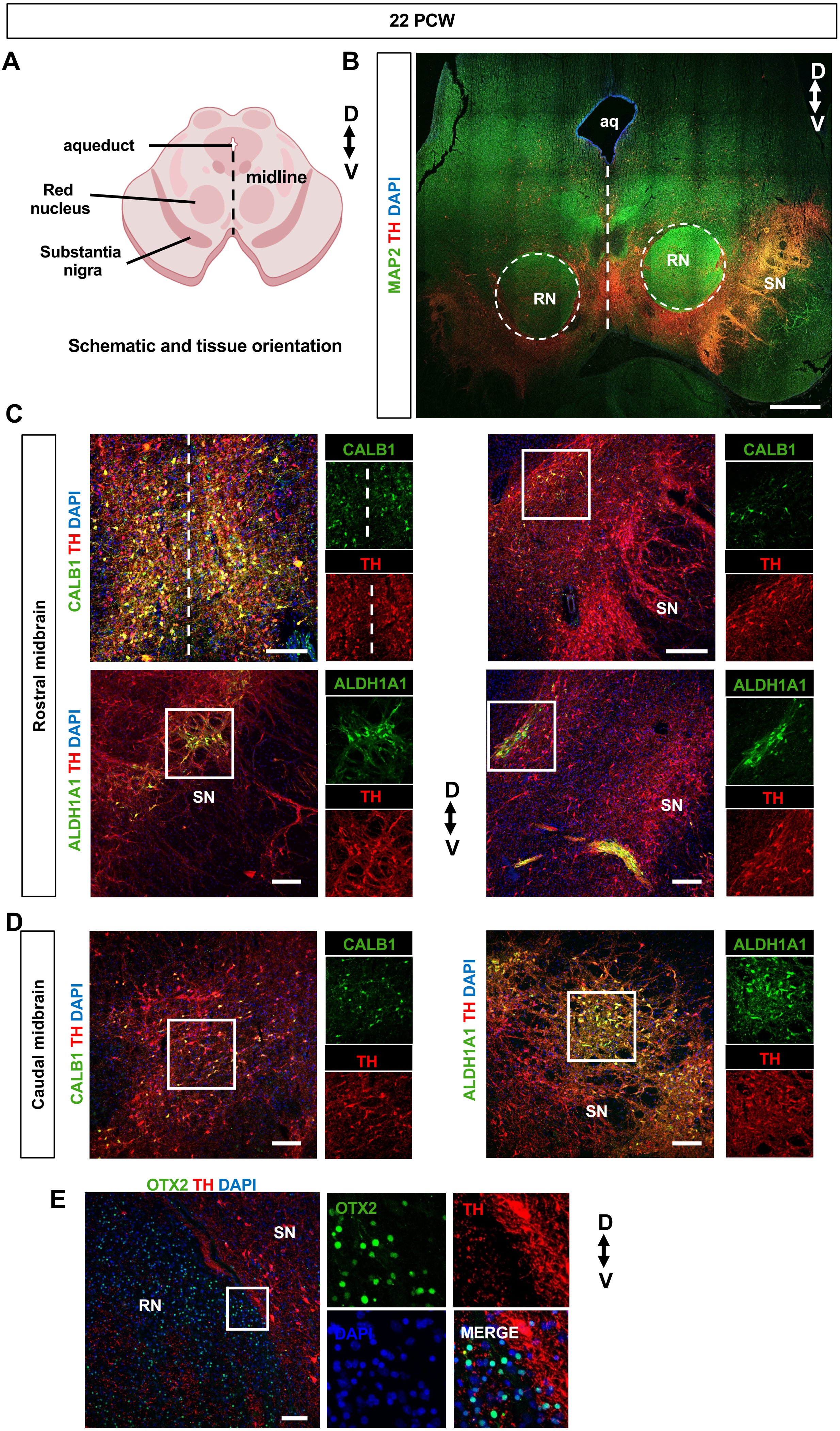
22 PCW
A
aqueduct
midline
Red nucleus
Substantia nigra
Schematic and tissue orientation
B
D
V
MAP2 TH DAPI
aq
RN
RN
SN
C
Rostral midbrain
CALB1 TH DAPI
CALB1
TH
SN
ALDH1A1 TH DAPI
ALDH1A1
SN
D
Caudal midbrain
CALB1 TH DAPI
ALDH1A1 TH DAPI
E
OTX2 TH DAPI
SN
RN
OTX2
TH
DAPI
MERGE

Supplementary Fig 4 Ventral midbrain dopaminergic neurons subtypes distribution at 22 PCW. A, Graphical representation of a coronal section of the human midbrain at 22 PCW. Created in BioRender. BUDINGER, D. (2025) https://BioRender.com/5cmho7m. **B**, Immunofluorescence analysis of human fetal sample at 19 PCW for MAP2 and TH. Scale bar = 300 µm. **C-D**, Representative images of immunostaining for CALB1, ALDH1A1 and TH in the rostral and caudal ventral midbrain at 22 PCW, respectively. Scale bars = 100 µm. **E**, Immunofluorescence analysis of OTX2 and TH expression. Scale bar = 100 µm. Nuclei are stained for DAPI. Lines demarcate ventral midline. Aqueduct (aq), dorsal (D), ventral (V), substantia nigra (SN), red nucleus (RN).

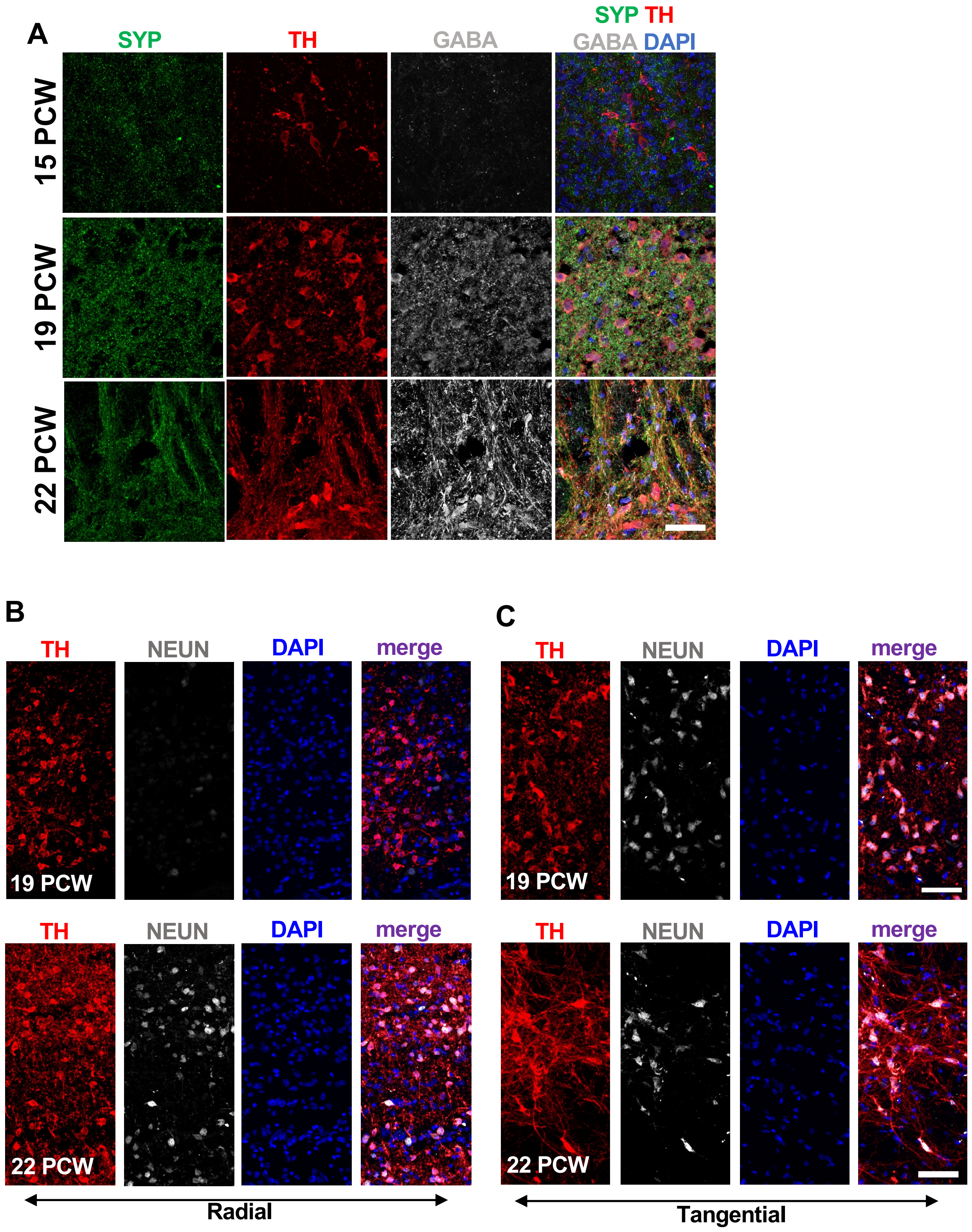
A
SYP
TH
GABA
SYP TH
GABA DAPI
15 PCW
19 PCW
22 PCW
B
C
TH
NEUN
DAPI
merge
19 PCW
22 PCW
Radial
Tangential

Supplementary Fig 5 Human midbrain dopaminergic neurons subtypes and maturation stage. A, Immunofluorescence analysis for Synaptophisin (SYP), TH and GABA of fetal samples at 15, 19 and 22 PCW. **B-C**, Representative immunofluorescence images of radial and tangential migrating dopaminergic neurons (TH+) and maturation stage as indicated by NEUN positivity. Nuclei are stained for DAPI. Scale bars = 50 μm.

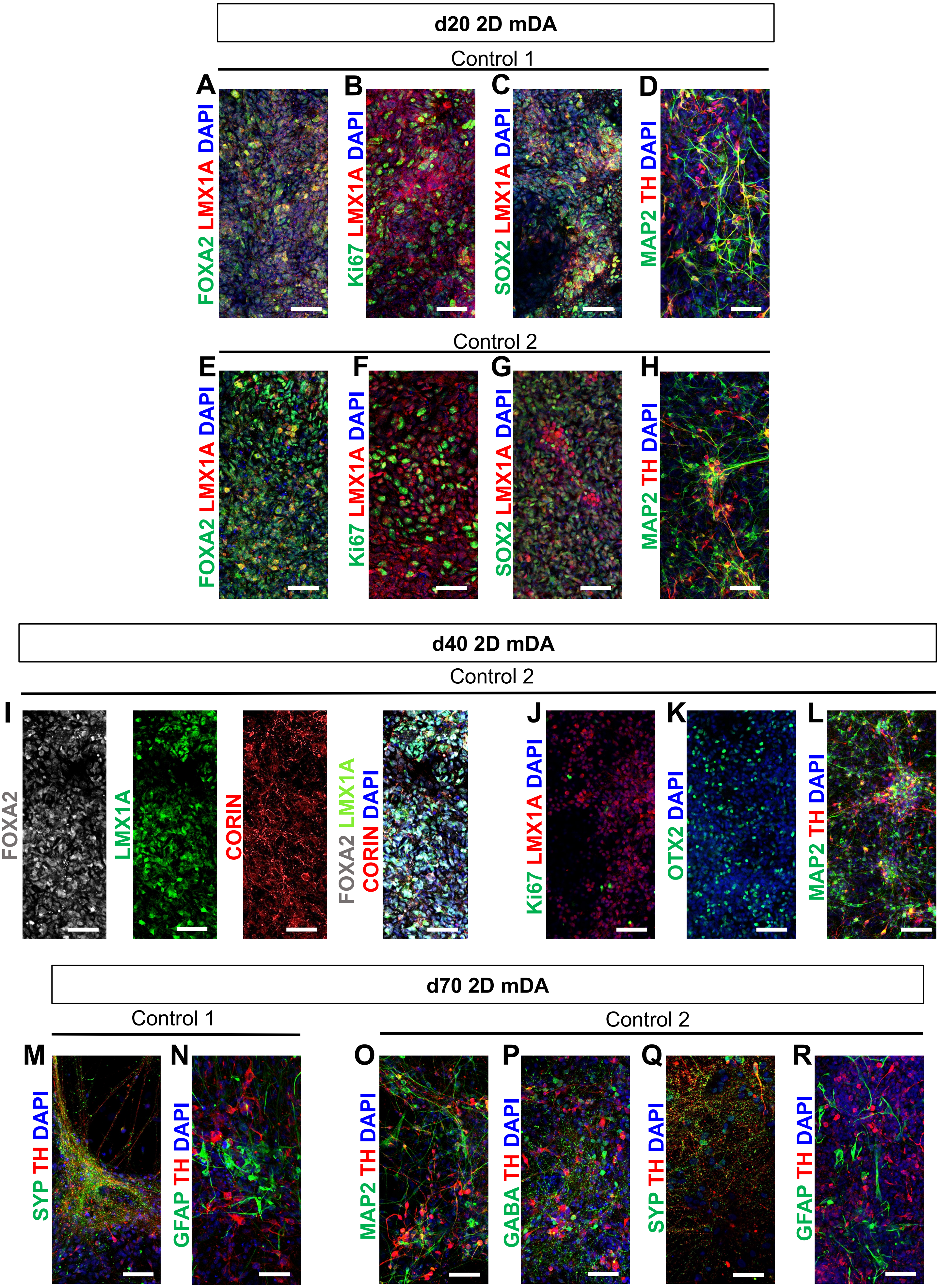

d20 2D mDA
Control 1
A
FOXA2 LMX1A DAPI
B
Ki67 LMX1A DAPI
C
SOX2 LMX1A DAPI
D
MAP2 TH DAPI
Control 2
E
FOXA2 LMX1A DAPI
F
Ki67 LMX1A DAPI
G
SOX2 LMX1A DAPI
H
MAP2 TH DAPI
d40 2D mDA
Control 2
I
FOXA2
LMX1A
CORIN
FOXA2 LMX1A CORIN DAPI
J
Ki67 LMX1A DAPI
K
OTX2 DAPI
L
MAP2 TH DAPI
d70 2D mDA
Control 1
Control 2
M
SYP TH DAPI
N
GFAP TH DAPI
O
MAP2 TH DAPI
P
GABA TH DAPI
Q
SYP TH DAPI
R
GFAP TH DAPI

Supplementary Fig 6 Characterization of iPSC-derived 2D mDA neuronal cultures. A-R, Representative images of immunofluorescence analysis for midbrain and neuronal-related proteins FOXA2, LMX1A, CORIN, SOX2, MAP2, TH, OTX2, GABA, SYP, the glial cell marker GFAP and the cell cycle protein Ki67 in Control 1 and Control 2-derived 2D mDA neural cultures at 20, 40 and 70 days of differentiation. Nuclei are staining with DAPI. Scale bars = 50 μm.

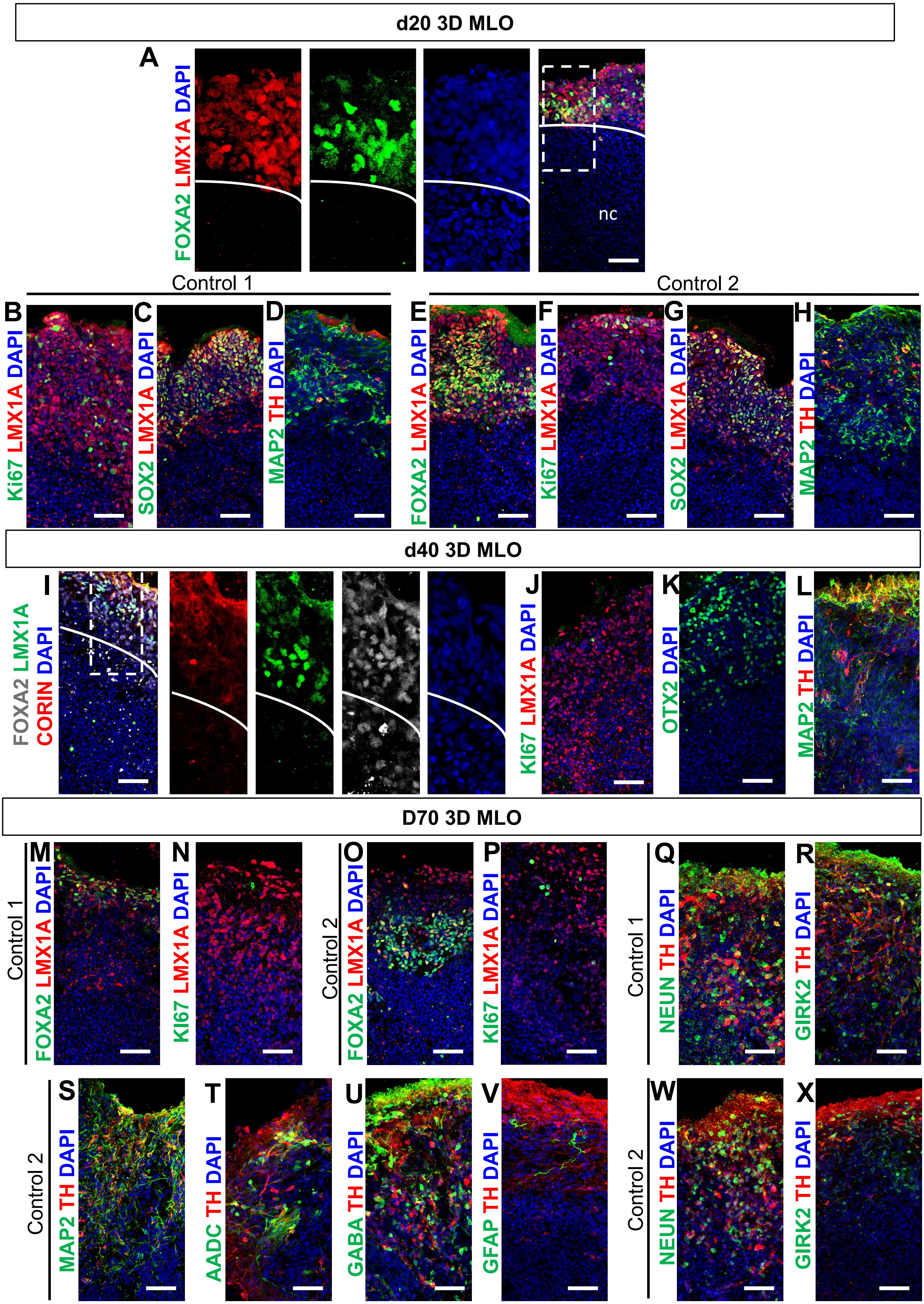
d20 3D MLO
A
FOXA2 LMX1A DAPI
nc
Control 1
Control 2
B
Ki67 LMX1A DAPI
C
SOX2 LMX1A DAPI
D
MAP2 TH DAPI
E
FOXA2 LMX1A DAPI
F
Ki67 LMX1A DAPI
G
SOX2 LMX1A DAPI
H
MAP2 TH DAPI
d40 3D MLO
I
FOXA2 LMX1A CORIN DAPI
J
KI67 LMX1A DAPI
K
OTX2 DAPI
L
MAP2 TH DAPI
D70 3D MLO
M
Control 1
FOXA2 LMX1A DAPI
N
KI67 LMX1A DAPI
O
Control 2
FOXA2 LMX1A DAPI
P
KI67 LMX1A DAPI
Q
Control 1
NEUN TH DAPI
R
GIRK2 TH DAPI
S
Control 2
MAP2 TH DAPI
T
AADC TH DAPI
U
GABA TH DAPI
V
GFAP TH DAPI
W
Control 2
NEUN TH DAPI
X
GIRK2 TH DAPI

Supplementary Fig 7 Characterization of iPSC-derived MLOs. A-X, Representative images of immunofluorescence analysis for midbrain and neuronal-related proteins FOXA2, LMX1A, CORIN, SOX2, MAP2, TH, AADC, OTX2, GABA, NEUN, GIRK2, the glial cell marker GFAP and the cell cycle protein Ki67 in Control 1 and Control 2-derived MLO at 20, 40 and 70 days of differentiation. Nuclei are staining with DAPI. Scale bars = 50 μm.

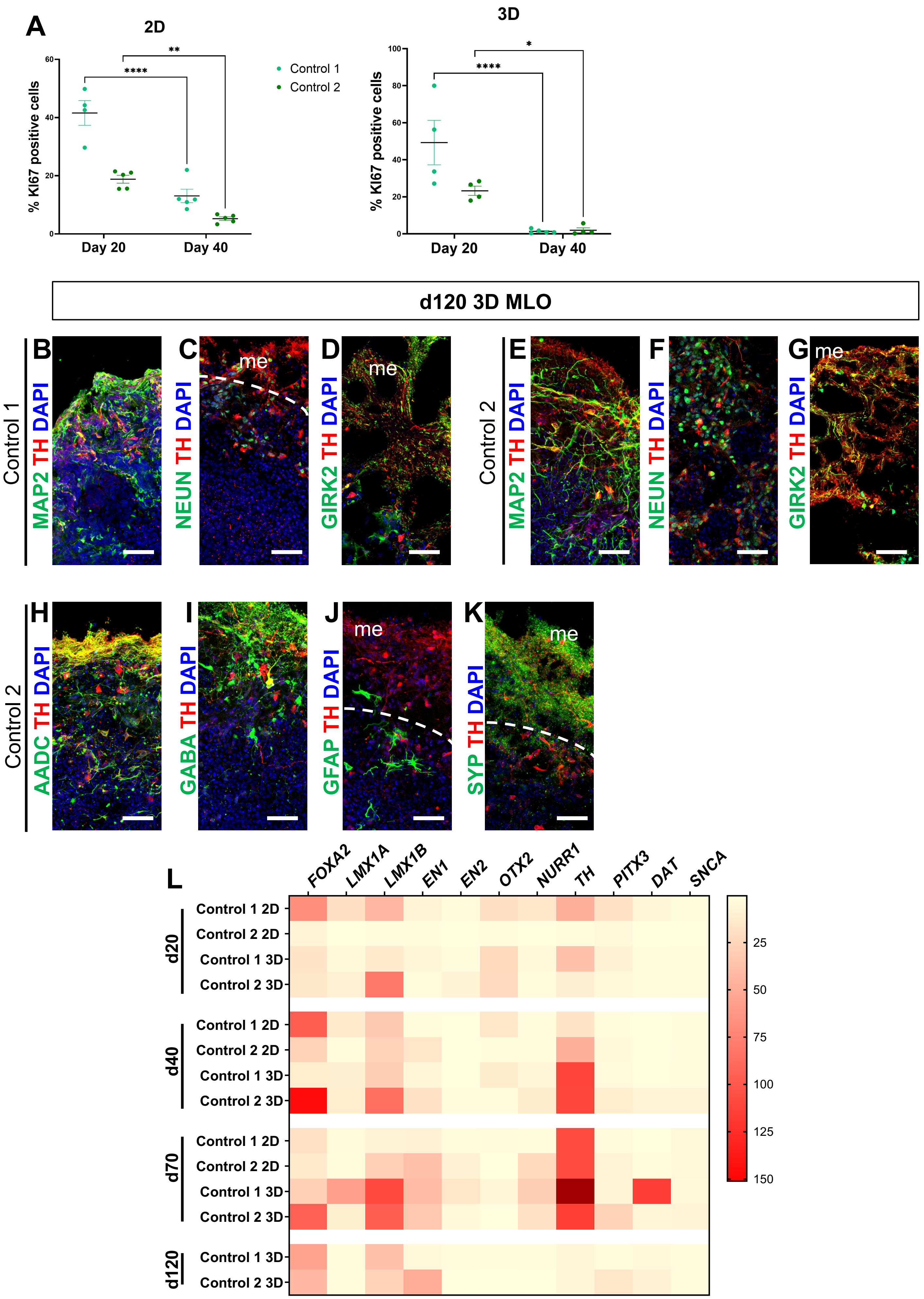
A
2D
3D
% KI67 positive cells
Day 20
Day 40
Control 1
Control 2
d120 3D MLO
B C D E F G H I J K
Control 1
Control 2
MAP2 TH DAPI
NEUN TH DAPI
GIRK2 TH DAPI
AADC TH DAPI
GABA TH DAPI
GFAP TH DAPI
SYP TH DAPI
me
L
FOXA2 LMX1A LMX1B EN1 EN2 OTX2 NURR1 TH PITX3 DAT SNCA
d20
d40
d70
d120
Control 1 2D
Control 2 2D
Control 1 3D
Control 2 3D
25
50
75
100
125
150

Supplementary Fig 8 Characterization of iPSC-derived MLO at late stage of maturation. A, Quantification for KI67+ cells in controls in both 2D cultures and 3D organoids, between d20 and d40 of differentiation. **B-K**, Immunofluorescence images of Control 1 and 2-derived MLO at 120 days of differentiation showing positive cells for TH, MAP2, NEUN, GIRK2, GFAP and SYP. Nuclei are staining with DAPI. Scale bars = 50 µm. **L**, Quantitative real time PCR (q-RT-PCR) for midbrain related genes in Control 1 and 2-derived 2D mDA (2D) cultures and MLOs at 20, 40, 70 and 120 days of differentiation. Genes expression for *FOXA2*, *LMX1A*, *LMX1B*, *EN1*, *EN2*, *OTX2*, *NURR1*, *PITX3*, *DAT*, *SNCA* is relative to housekeeping gene (*GAPDH*) and normalized to their respective iPSCs (n=1 for each line). Error bars indicate SEM. 2D and 3D cultures at day 20 and day 40 were independently compared using two-tailed Student's *t*-test for all analyses. Matrigel embedding (me). Source data are provided as a Source Data file.

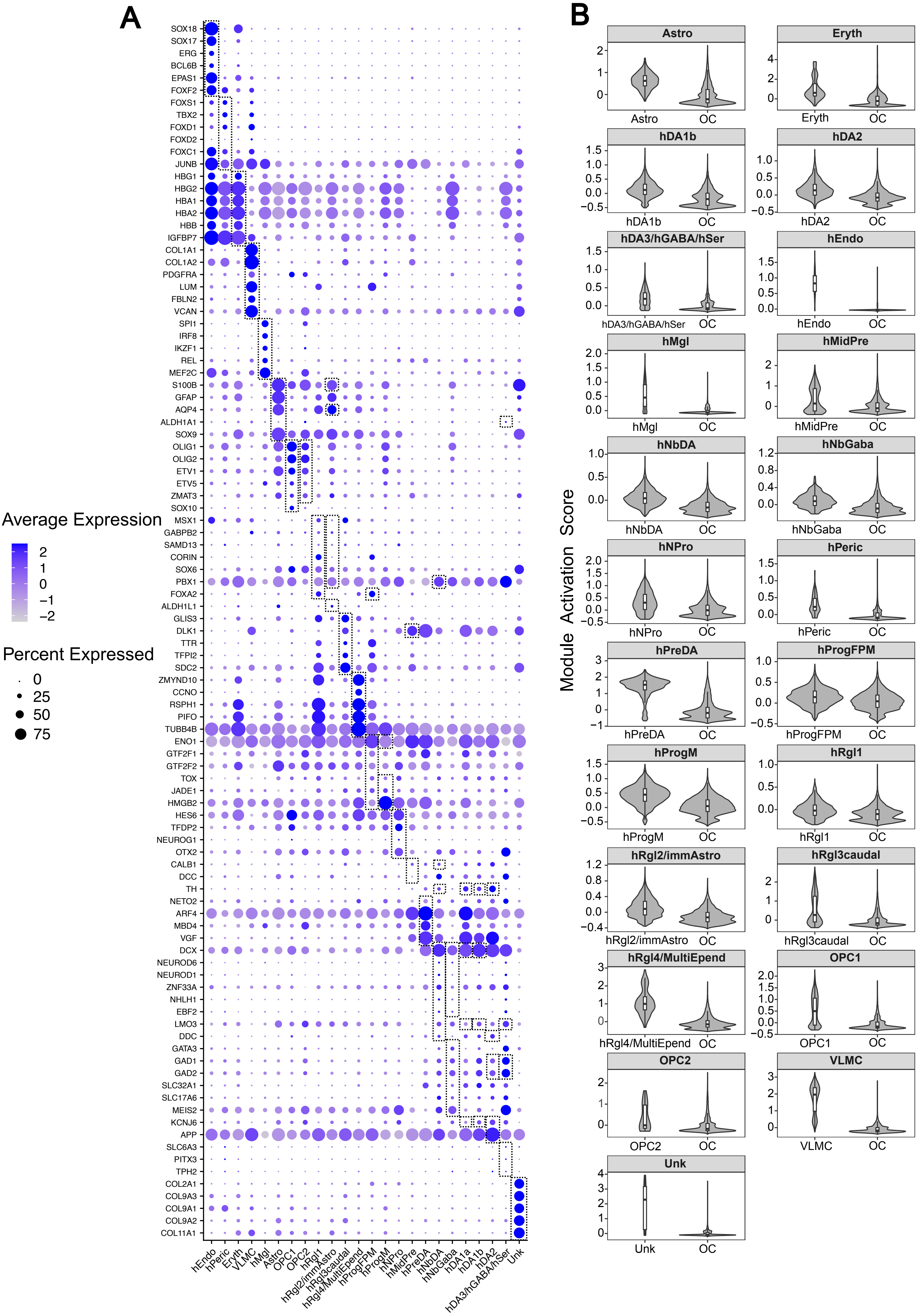
A
B
Average Expression
Percent Expressed
Module Activation Score
Astro
Eryth
hDA1b
hDA2
hDA3/hGABA/hSer
hEndo
hMgl
hMidPre
hNbDA
hNbGaba
hNPro
hPeric
hPreDA
hProgFPM
hProgM
hRgl1
hRgl2/immAstro
hRgl3caudal
hRgl4/MultiEpend
OPC1
OPC2
VLMC
Unk
OC

Supplementary Fig 9 High-resolution cell type annotation based on marker genes and gene modules. A, Marker genes used for high-resolution cell type annotation. The blue gradient indicates the scaled average expression per cell type, while the dot size indicates the percentage of cells expressing each gene within the corresponding cell type. For further details on marker genes and abbreviations, see **Supp Data S3**. **B**, Violin plots showing the distribution of activation score for cell-type specific gene modules (Supp Data S3), comparing the cell type of interest to other cell types (OC).

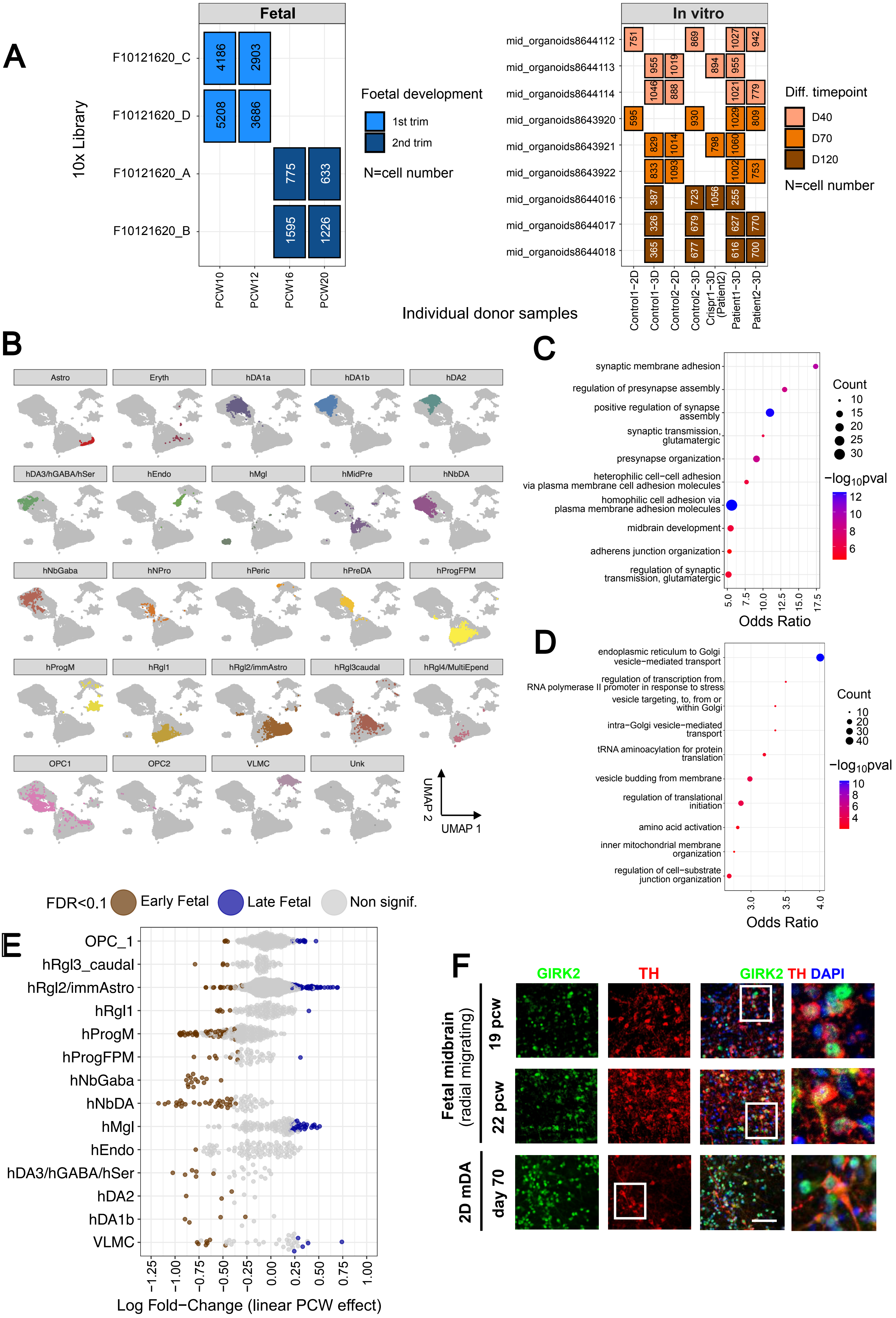
A
Fetal
In vitro
10x Library
F10121620_C
F10121620_D
F10121620_A
F10121620_B
4186
2903
5208
3686
775
633
1595
1226
PCW10
PCW12
PCW16
PCW20
Foetal development
1st trim
2nd trim
N=cell number
mid_organoids8644112
mid_organoids8644113
mid_organoids8644114
mid_organoids8643920
mid_organoids8643921
mid_organoids8643922
mid_organoids8644016
mid_organoids8644017
mid_organoids8644018
751
869
1027
942
955
1019
894
955
1046
888
1021
779
595
930
1029
809
829
1014
798
1060
833
1093
1002
753
387
723
1056
255
326
679
627
770
365
677
616
700
Control1−2D
Control1−3D
Control2−2D
Control2−3D
Crispr1−3D (Patient2)
Patient1−3D
Patient2−3D
Individual donor samples
Diff. timepoint
D40
D70
D120
N=cell number
B
Astro
Eryth
hDA1a
hDA1b
hDA2
hDA3/hGABA/hSer
hEndo
hMgl
hMidPre
hNbDA
hNbGaba
hNPro
hPeric
hPreDA
hProgFPM
hProgM
hRgl1
hRgl2/immAstro
hRgl3caudal
hRgl4/MultiEpend
OPC1
OPC2
VLMC
Unk
UMAP 2
UMAP 1
C
synaptic membrane adhesion
regulation of presynapse assembly
positive regulation of synapse assembly
synaptic transmission, glutamatergic
presynapse organization
heterophilic cell−cell adhesion via plasma membrane cell adhesion molecules
homophilic cell adhesion via plasma membrane adhesion molecules
midbrain development
adherens junction organization
regulation of synaptic transmission, glutamatergic
5.0
7.5
10.0
12.5
15.0
17.5
Odds Ratio
Count
10
15
20
25
30
−log10pval
12
10
8
6
D
endoplasmic reticulum to Golgi vesicle−mediated transport
regulation of transcription from RNA polymerase II promoter in response to stress
vesicle targeting, to, from or within Golgi
intra−Golgi vesicle−mediated transport
tRNA aminoacylation for protein translation
vesicle budding from membrane
regulation of translational initiation
amino acid activation
inner mitochondrial membrane organization
regulation of cell−substrate junction organization
3.0
3.5
4.0
Odds Ratio
Count
10
20
30
40
−log10pval
10
8
6
4
E
FDR<0.1
Early Fetal
Late Fetal
Non signif.
OPC_1
hRgl3_caudal
hRgl2/immAstro
hRgl1
hProgM
hProgFPM
hNbGaba
hNbDA
hMgl
hEndo
hDA3/hGABA/hSer
hDA2
hDA1b
VLMC
−1.25
−1.00
−0.75
−0.50
−0.25
0.00
0.25
0.50
0.75
1.00
Log Fold−Change (linear PCW effect)
F
GIRK2
TH
GIRK2 TH DAPI
Fetal midbrain (radial migrating)
19 pcw
22 pcw
2D mDA
day 70

Supplementary Fig 10 Single cell RNA-seq profiling of in vitro and in vivo fetal samples. A, Dataset overview (this study) showing donor/model combinations for fetal (left) and in vitro (2D/3D, right) samples. Each column represents a donor/model with 2-9 replicates, totaling 1,346-9,394 cells. **B**, UMAP visualizations by high-resolution cell type annotation, with each facet showing one cell type: astrocytes (Astro), erythrocytes (Eryth), dopaminergic neurons (hDA1a, hDA1b, hDA2), mixed group of dopaminergic, GABAergic-related and serotonergic neurons (hDA3/hGABA/hSer), endothelial cells (hEndo), microglia (hMgl), midbrain precursors (hMidPre), dopaminergic neuroblasts (hNbDA), GABAergic neuroblasts (hNbGaba), neuronal progenitors (hNPro), pericytes (hPeric), dopaminergic precursors (hPreDA), progenitors medial floorplate (hProgFPM), progenitor midline (hProgM), radial glia 1 (hRgl1), radial glia 2 / immature astrocytes (hRgl2/immAstro), radial glia 3 (hRgl3caudal), radial glia 4/ependymal cells (hRgl4/MultiEpend), oligodendrocytes progenitor cells (OPC1, OPC2), vascular leptomeningeal cells (VLMC) and unknown cells (Unk). **C**, Upregulation of synaptic-related pathways in fetal vs 3D hDA2 cells. Only the top-10 upregulated biological processes are shown (gene ontology enrichment parameters: pval<0.05, minSize=25, maxSize=500, minCount=10). **D**, Downregulation of transcription and translation-related pathways in fetal vs 3D hDA2 cells. Only the top-10 downregulated biological processes are shown (gene ontology enrichment parameters: pval<0.05, minSize=25, maxSize=500, minCount=10). **E,** Differential abundance analysis between early and late fetal cell types. Microglia is enriched in second trimester samples, while immature neurons (hNbGaba, hNbDA) and dopaminergic neurons (hDA2, hDA1b) are more abundant in the first trimester. Some cell types (ie, OPC1, hRgl2/immAstro) show enrichment at both stages, reflecting transitional states. **F**, Immunofluorescence analysis of fetal midbrain at 19 and 22 PCW and MLOs at 70 and 120 days of differentiation for TH and the potassium channel GIRK2. Nuclei are staining with DAPI. Scale bar = 50 µm.

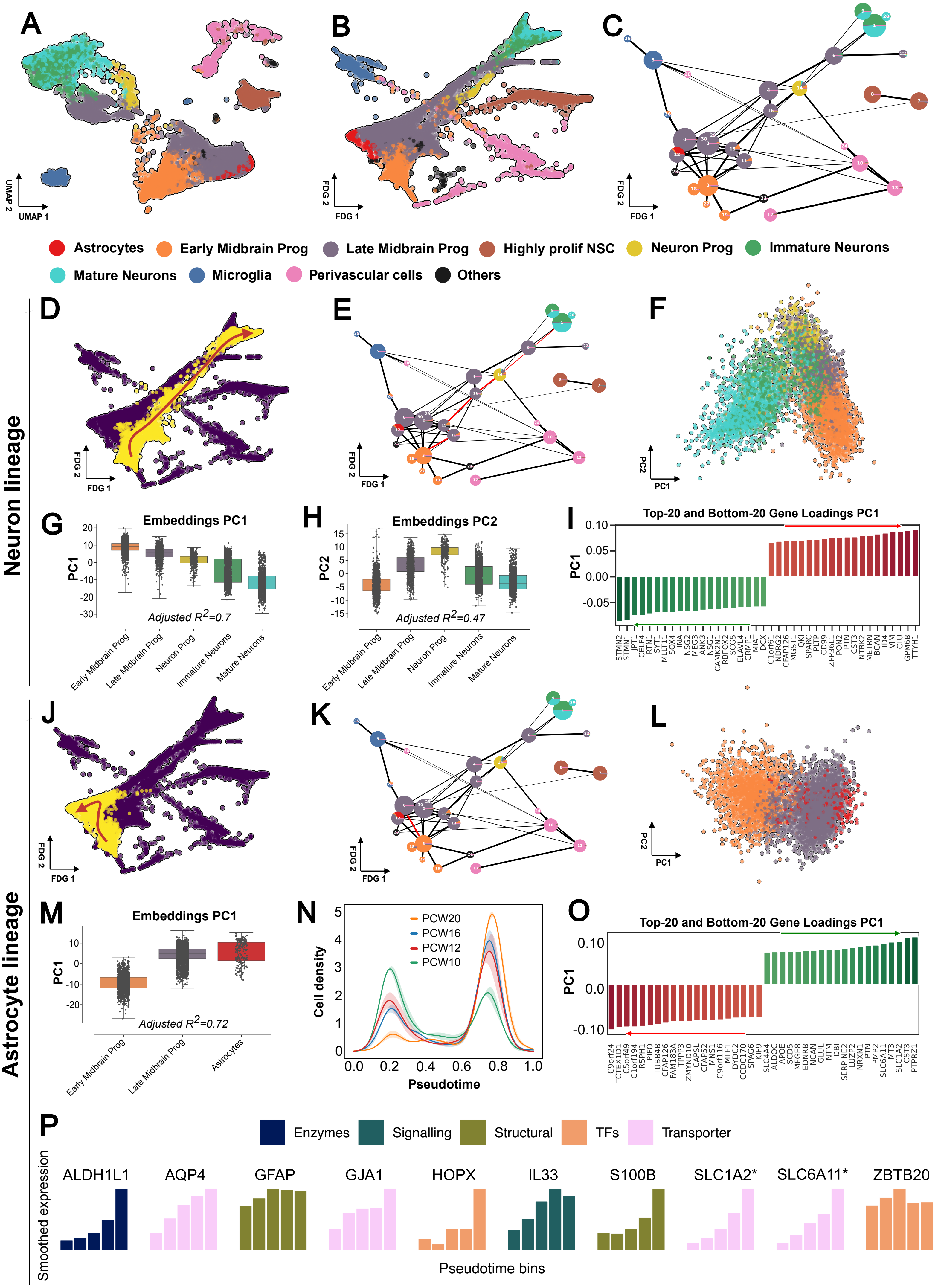

A
B
C
UMAP 1
UMAP 2
FDG 1
FDG 2
Astrocytes
Early Midbrain Prog
Late Midbrain Prog
Highly prolif NSC
Neuron Prog
Immature Neurons
Mature Neurons
Microglia
Perivascular cells
Others
Neuron lineage
D
E
F
PC1
PC2
G
Embeddings PC1
Adjusted R^2=0.7
H
Embeddings PC2
Adjusted R^2=0.47
I
Top-20 and Bottom-20 Gene Loadings PC1
Early Midbrain Prog
Late Midbrain Prog
Neuron Prog
Immature Neurons
Mature Neurons
Astrocyte lineage
J
K
L
M
Embeddings PC1
Adjusted R^2=0.72
Astrocytes
N
PCW20
PCW16
PCW12
PCW10
Cell density
Pseudotime
O
Top-20 and Bottom-20 Gene Loadings PC1
P
Enzymes
Signalling
Structural
TFs
Transporter
ALDH1L1
AQP4
GFAP
GJA1
HOPX
IL33
S100B
SLC1A2*
SLC6A11*
ZBTB20
Smoothed expression
Pseudotime bins

Supplementary Fig 11 Trajectory analysis of neuronal and astrocyte lineages. A, UMAP visualization of fetal cells (PCW 10,12, 16, 20), coloured by low-resolution cell type annotation. **B,** Force-directed graph (FDG) representation of the same fetal cells. **C**, Partition-based graph abstraction (PAGA) showing the connectivity between Leiden clusters (resolution=1), with low-weight (w<0.05) edges pruned. **D-E**, Inferred neuronal trajectory with cells highlighted in yellow and pseudotime direction indicated with the red arrow (d), built from the shortest path connectivity (e) between the starting point (cluster 3, early midbrain progenitors) and the ending point (cluster 20, mature neurons). Path indicated with red edges. **F,** Principal component analysis (PCA) of the neuronal lineage cells, coloured by cell type. **G-H**, Correlation between PC1 (g) or PC2 (h) variance with the annotated neuronal cell types (adjusted r-square, p<0.05). Only PC1 captures the progression along the differentiation path. **I**, Top-20 and bottom-20 PC1 gene loadings, showing gene expression correlation with neuronal maturation (in green) or with progenitor-like state (in red). **J-K**, Inferred astrocyte trajectory with cells highlighted in yellow and pseudotime direction indicated with the red arrow (j), built from the path connectivity (k) between the starting point (cluster 3, early midbrain progenitors), the intermediate point (cluster 0, late midbrain progenitors) and the ending point (cluster 12, astrocytes). Path indicated with red edges. **L**, PCA of the astrocyte lineage cells, coloured by cell type. **M**, PC1 captures the progression along the astrocyte differentiation (adjusted R^2 correlation, p<0.05). **N**, Pseudotime density across fetal samples: solid lines show the replicate means (n=2); shaded area indicates to ± 1 SD among replicate densities. **O**, Top-20 and bottom-20 PC1 gene loadings, showing gene expression correlation with astrocyte maturation (in green). **P**, Smoothed expression profiles of a curated set of marker genes tracking the astrocyte lineage, across five evenly spaced pseudotime bins. Asterisks indicate significant start-to-end gene expression changes, as evaluated per tradeSeq. The color scheme of the 10 cell types (from the low-clustering resolution) is shared among panels **A-C**, **E-H** and **K-M**.

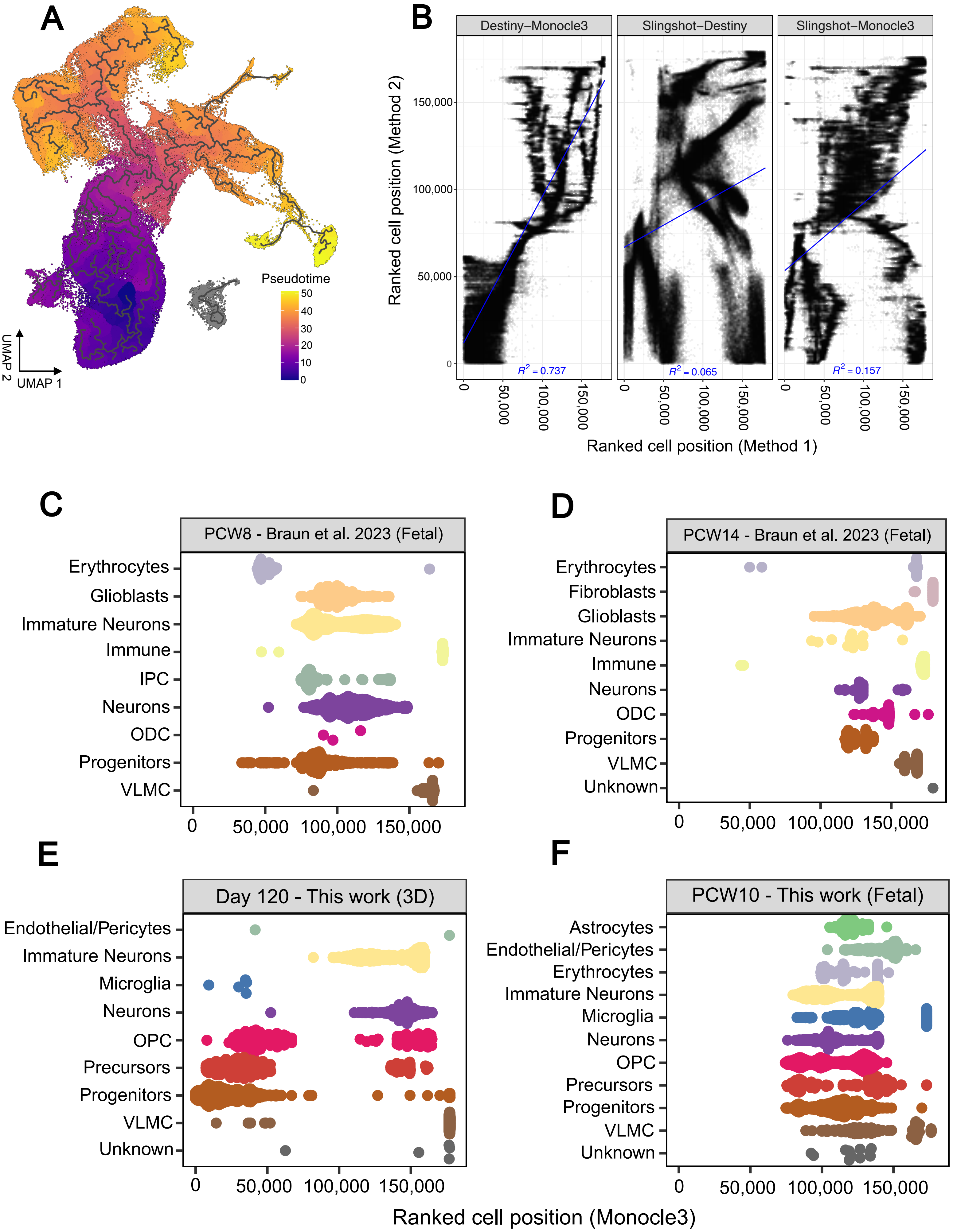

A
Pseudotime
50
40
30
20
10
0
UMAP 2
UMAP 1
B
Destiny–Monocle3
Slingshot–Destiny
Slingshot–Monocle3
Ranked cell position (Method 2)
150,000
100,000
50,000
0
R^2 = 0.737
R^2 = 0.065
R^2 = 0.157
Ranked cell position (Method 1)
C
PCW8 - Braun et al. 2023 (Fetal)
Erythrocytes
Glioblasts
Immature Neurons
Immune
IPC
Neurons
ODC
Progenitors
VLMC
0
50,000
100,000
150,000
D
PCW14 - Braun et al. 2023 (Fetal)
Erythrocytes
Fibroblasts
Glioblasts
Immature Neurons
Immune
Neurons
ODC
Progenitors
VLMC
Unknown
E
Day 120 - This work (3D)
Endothelial/Pericytes
Immature Neurons
Microglia
Neurons
OPC
Precursors
Progenitors
VLMC
Unknown
F
PCW10 - This work (Fetal)
Astrocytes
Endothelial/Pericytes
Erythrocytes
Immature Neurons
Microglia
Neurons
OPC
Precursors
Progenitors
VLMC
Unknown
Ranked cell position (Monocle3)

Supplementary Fig 12. Meta-integration of multiple dopaminergic scRNA-seq datasets and pseudotemporal dynamics analysis. A, Pseudotime trajectory inferred by Monocle3 on the UMAP projection. The gradient ranges from 0 (dark blue, rooted at floor plate progenitors, FPP) to 50 (yellow, corresponding to oligodendrocytes), capturing the trajectory of cells undergoing dopaminergic differentiation. **B**, Comparison of pseudotime trajectories: pairwise correlation of ranked cell position inferred by Monocle3, Slingshot and Destiny. Only Monocle3 and Destiny show a high degree of agreement (R^2=0.737). **C-F,** Violin scatter plots depicting Monocle3-inferred pseudotime distribution per cell type, based on the unified annotation for the integration. Panels correspond to post-conceptional week 8 (c) and 14 (d) from Braun et al. 2023; or to the 3D-MLO in vitro model (e) and the fetal post-conceptional week 10 (f) from this study. The x axis reflects the ranked cell position across the integrated dataset, used here as a pseudotime proxy.

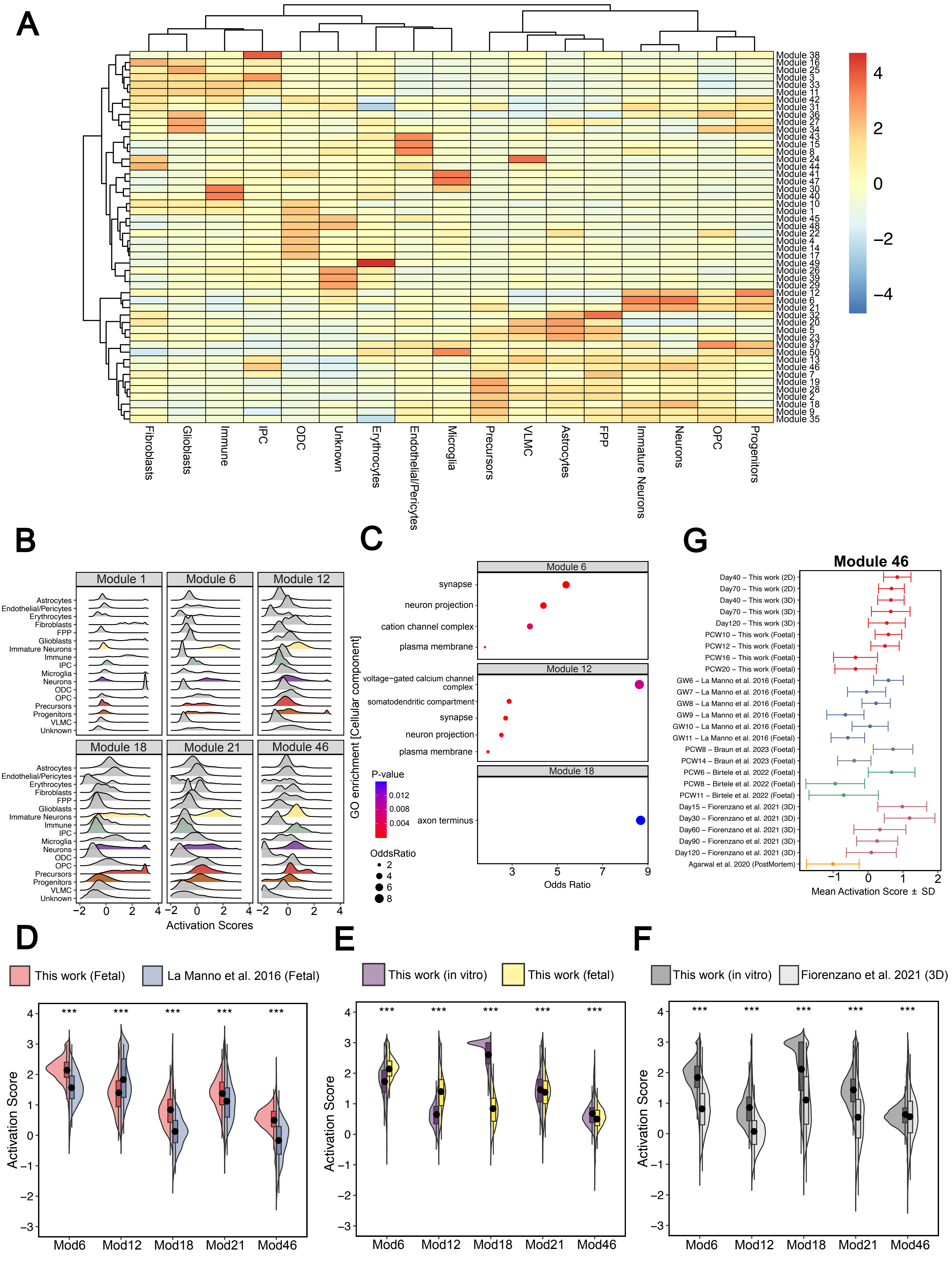
A
Module 38
Module 16
Module 25
Module 3
Module 33
Module 11
Module 42
Module 31
Module 36
Module 27
Module 34
Module 43
Module 15
Module 8
Module 24
Module 44
Module 41
Module 47
Module 30
Module 40
Module 10
Module 1
Module 45
Module 48
Module 22
Module 4
Module 14
Module 17
Module 49
Module 26
Module 39
Module 29
Module 12
Module 6
Module 21
Module 32
Module 20
Module 5
Module 23
Module 37
Module 50
Module 13
Module 46
Module 7
Module 19
Module 28
Module 2
Module 18
Module 9
Module 35
4
2
0
−2
−4
Fibroblasts
Glioblasts
Immune
IPC
ODC
Unknown
Erythrocytes
Endothelial/Pericytes
Microglia
Precursors
VLMC
Astrocytes
FPP
Immature Neurons
Neurons
OPC
Progenitors
B
Module 1
Module 6
Module 12
Module 18
Module 21
Module 46
Astrocytes
Endothelial/Pericytes
Erythrocytes
Fibroblasts
FPP
Glioblasts
Immature Neurons
Immune
IPC
Microglia
Neurons
ODC
OPC
Precursors
Progenitors
VLMC
Unknown
Activation Scores
C
GO enrichment [Cellular component]
Module 6
synapse
neuron projection
cation channel complex
plasma membrane
Module 12
voltage−gated calcium channel complex
somatodendritic compartment
synapse
neuron projection
plasma membrane
Module 18
axon terminus
P-value
0.012
0.008
0.004
OddsRatio
2
4
6
8
Odds Ratio
G
Module 46
Day40 – This work (2D)
Day70 – This work (2D)
Day40 – This work (3D)
Day70 – This work (3D)
Day120 – This work (3D)
PCW10 – This work (Foetal)
PCW12 – This work (Foetal)
PCW16 – This work (Foetal)
PCW20 – This work (Foetal)
GW6 – La Manno et al. 2016 (Foetal)
GW7 – La Manno et al. 2016 (Foetal)
GW8 – La Manno et al. 2016 (Foetal)
GW9 – La Manno et al. 2016 (Foetal)
GW10 – La Manno et al. 2016 (Foetal)
GW11 – La Manno et al. 2016 (Foetal)
PCW8 – Braun et al. 2023 (Foetal)
PCW14 – Braun et al. 2023 (Foetal)
PCW6 – Birtele et al. 2022 (Foetal)
PCW8 – Birtele et al. 2022 (Foetal)
PCW11 – Birtele et al. 2022 (Foetal)
Day15 – Fiorenzano et al. 2021 (3D)
Day30 – Fiorenzano et al. 2021 (3D)
Day60 – Fiorenzano et al. 2021 (3D)
Day90 – Fiorenzano et al. 2021 (3D)
Day120 – Fiorenzano et al. 2021 (3D)
Agarwal et al. 2020 (PostMortem)
Mean Activation Score ± SD
D
This work (Fetal)
La Manno et al. 2016 (Fetal)
Activation Score
***
Mod6
Mod12
Mod18
Mod21
Mod46
E
This work (in vitro)
This work (fetal)
Activation Score
F
This work (in vitro)
Fiorenzano et al. 2021 (3D)
Activation Score

Supplementary Fig 13. Modules of co-regulated genes that change as a function of pseudotime. **A**, Heatmap displaying the activation scores of pseudotime-dependent gene modules across cell types. Each tile represents the average activation score (aggregated module scores) for each cell type (unified annotation in the columns). The inferred gene modules (rows, n=50) include groups of trajectory-variable genes that are co-regulated. **B**, Ridge density plot showing the activation scores across cell types for the ODC-specific module (Module 1) and the top-enriched modules in neurons (Modules 6, 12, 18, 21 and 46). Neuron-enriched modules are typically also activated in immature neurons and other cell types, such as progenitors (Modules 6 and 12), precursors (Module 18), and intermediate progenitor cells (Module 46). **C**, Gene ontology enrichment analysis for neuron-enriched modules (adjusted $p<0.05$). Module 6 is enriched in synapse function, module 12 in voltage-gated calcium channel complexes, and module 18 in axonal structure. Modules 21 and 46 showed no significant enrichment in gene ontology (GO) or KEGG pathways. Odds ratios were calculated based on term and query sizes, effective domain size and intersection size. **D**-**F**, Split violin plots comparing the activation scores for five neuron-enriched modules across datasets: fetal cells from this study vs. La Manno et al. 2016 (**D**); *in vitro* cells vs. fetal cells both from this study (**E**); and *in vitro* cells from this study vs. the 3D-dopaminergic model from Fiorenzano et al. 2021 (**F**), respectively. Significance level: p<10-3 (***, One-way permutation test). **G**, Activation score distribution for the neuron-enriched module 46 across timepoints (rows) and datasets (colors). Mean values and standard deviation error bars shown for each timepoint combination.

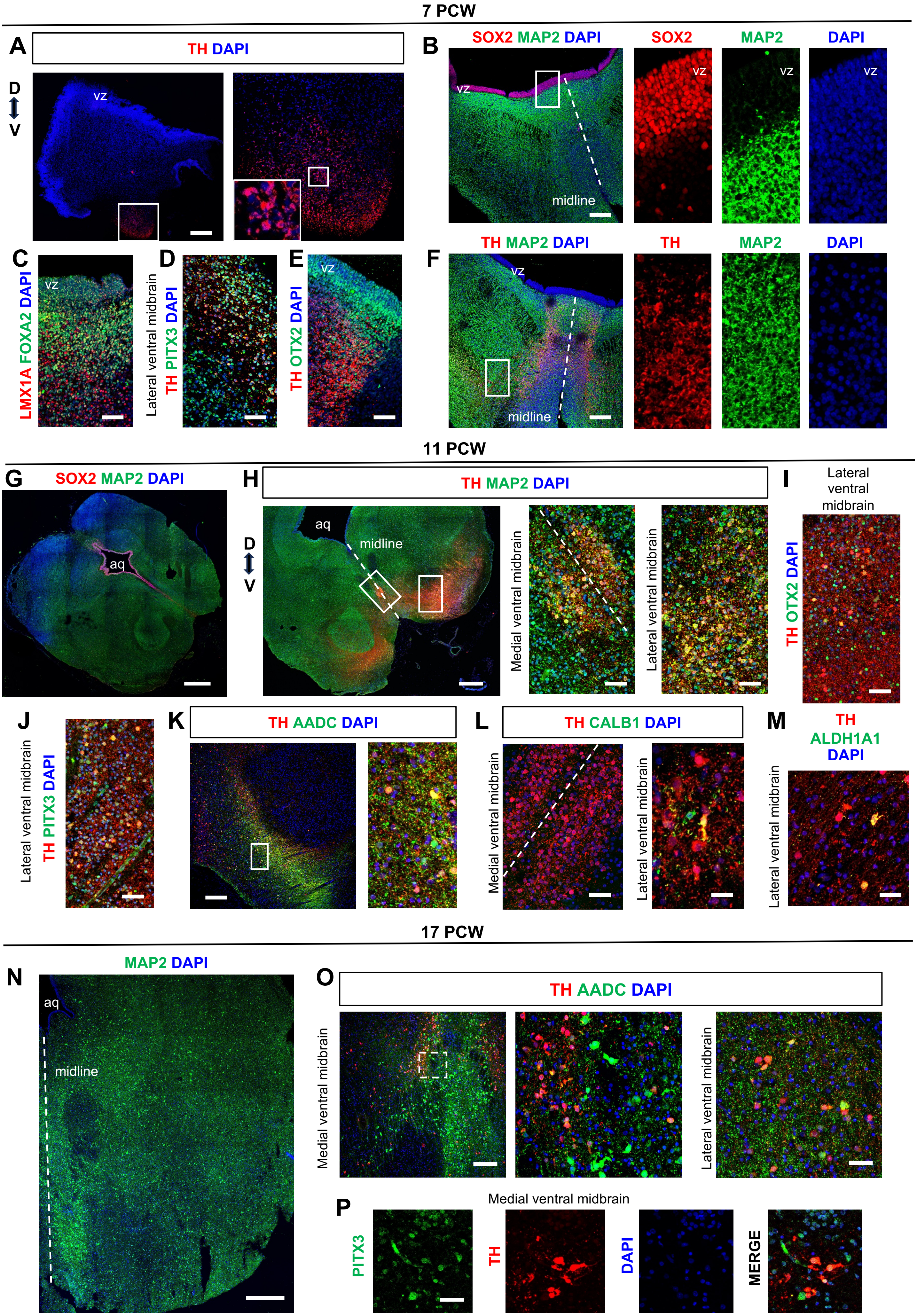

7 PCW
A
TH DAPI
D
V
vz
B
SOX2 MAP2 DAPI
SOX2
MAP2
DAPI
midline
C
LMX1A FOXA2 DAPI
D
Lateral ventral midbrain
TH PITX3 DAPI
E
TH OTX2 DAPI
F
TH MAP2 DAPI
TH
MAP2
DAPI
11 PCW
G
SOX2 MAP2 DAPI
aq
H
TH MAP2 DAPI
midline
Medial ventral midbrain
Lateral ventral midbrain
I
Lateral ventral midbrain
TH OTX2 DAPI
J
TH PITX3 DAPI
K
TH AADC DAPI
L
TH CALB1 DAPI
M
TH ALDH1A1 DAPI
17 PCW
N
MAP2 DAPI
O
TH AADC DAPI
P
Medial ventral midbrain
PITX3
TH
DAPI
MERGE

Supplementary Fig 14 Midbrain fetal systems. A-F Immunofluorescence images of 7 PCW fetal midbrain showing positive cells for TH (sample used in spatial analysis) (scale bar = 300 µm), SOX2 and MAP2 (scale bar = 200 µm), LMX1A and FOXA2 (scale bar = 50 µm), TH and PITX3 (scale bar = 50 µm), TH and OTX2 (scale bar = 50 µm), TH and MAP2 (scale bar = 200 µm). **G-M**, Immunofluorescence analysis of 11 PCW fetal midbrain for SOX2 and MAP2 (scale bar = 500 µm), TH and MAP2 (scale bar = 300 µm – *left panel*; scale bar = 100 µm – *right panel*), TH and OTX2 (scale bar = 50 µm), TH and PITX3 (scale bar = 50 µm), TH and AADC (scale bar = 200 µm), TH and CALB1 (scale bar = 100 µm – *left panel*; scale bar = 20 µm – *right panel*), TH and ALDH1A1 (scale bar = 20 µm). **O-P**, Representative immunofluorescence images of the 17 PCW human midbrain showing expression of MAP2 (scale bar = 500 µm), TH and AADC and TH (scale bar = 100 µm – *left panel*; scale bar = 50 µm – *right panel*) and PITX3 (scale bar = 30 µm) Nuclei are staining with DAPI. Ventricular zone (VZ), aqueduct (aq).

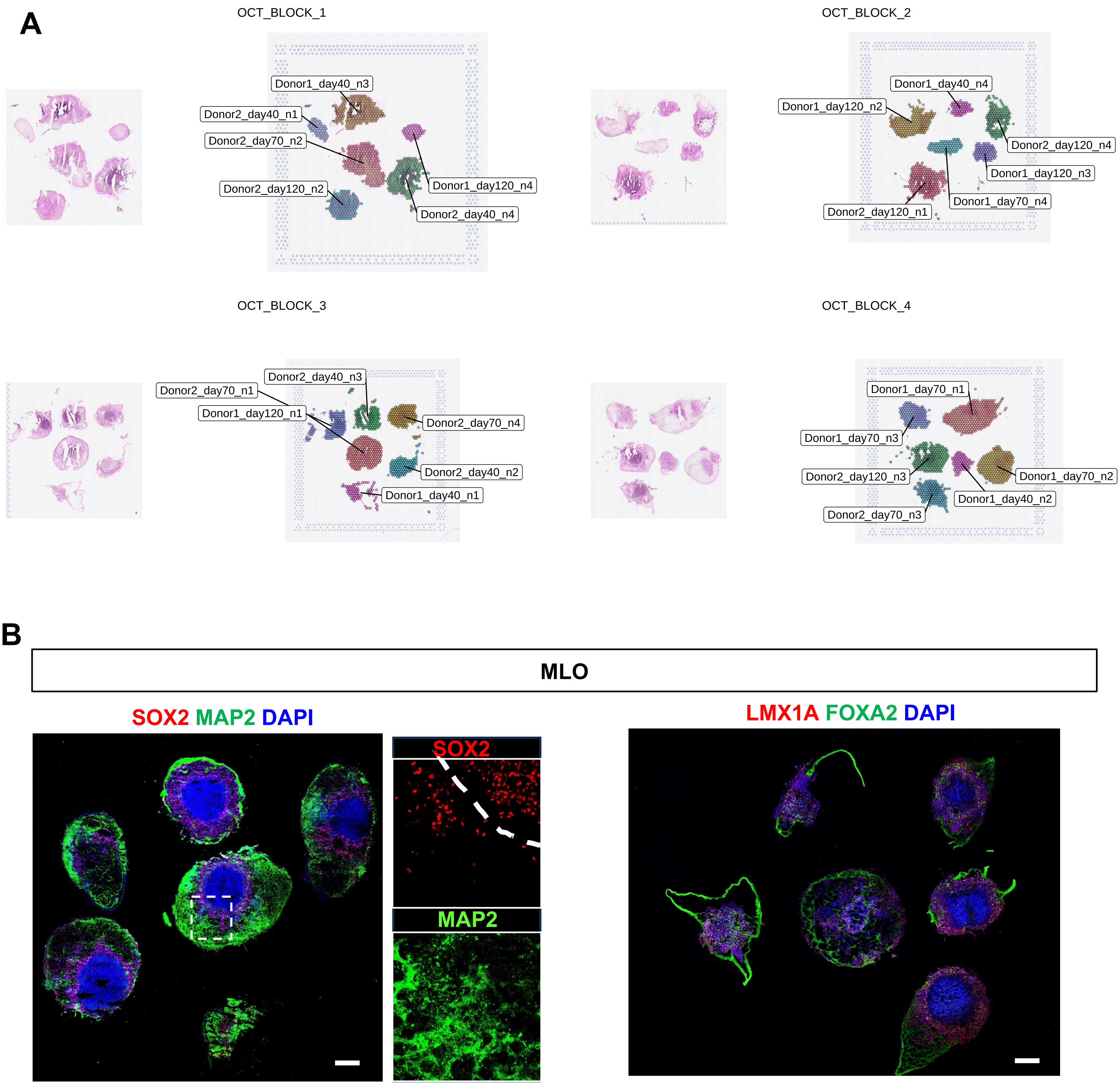
A
OCT_BLOCK_1
Donor1_day40_n3
Donor2_day40_n1
Donor2_day70_n2
Donor2_day120_n2
Donor1_day120_n4
Donor2_day40_n4
OCT_BLOCK_2
Donor1_day40_n4
Donor1_day120_n2
Donor2_day120_n4
Donor1_day120_n3
Donor1_day70_n4
Donor2_day120_n1
OCT_BLOCK_3
Donor2_day40_n3
Donor2_day70_n1
Donor1_day120_n1
Donor2_day70_n4
Donor2_day40_n2
Donor1_day40_n1
OCT_BLOCK_4
Donor1_day70_n1
Donor1_day70_n3
Donor2_day120_n3
Donor1_day70_n2
Donor1_day40_n2
Donor2_day70_n3
B
MLO
SOX2 MAP2 DAPI
SOX2
MAP2
LMX1A FOXA2 DAPI

Supplementary Fig 15 MLO systems. A, Arrangement of MLOs in Visium capture areas. **B**, Immunofluorescence analysis for SOX2, MAP2 (*left panel*), and LMX1A and FOXA2 (*right panel*) in MLO samples analyzed with spatial transcriptomic. Nuclei are stained with DAPI. Scale bars = 500 μm.

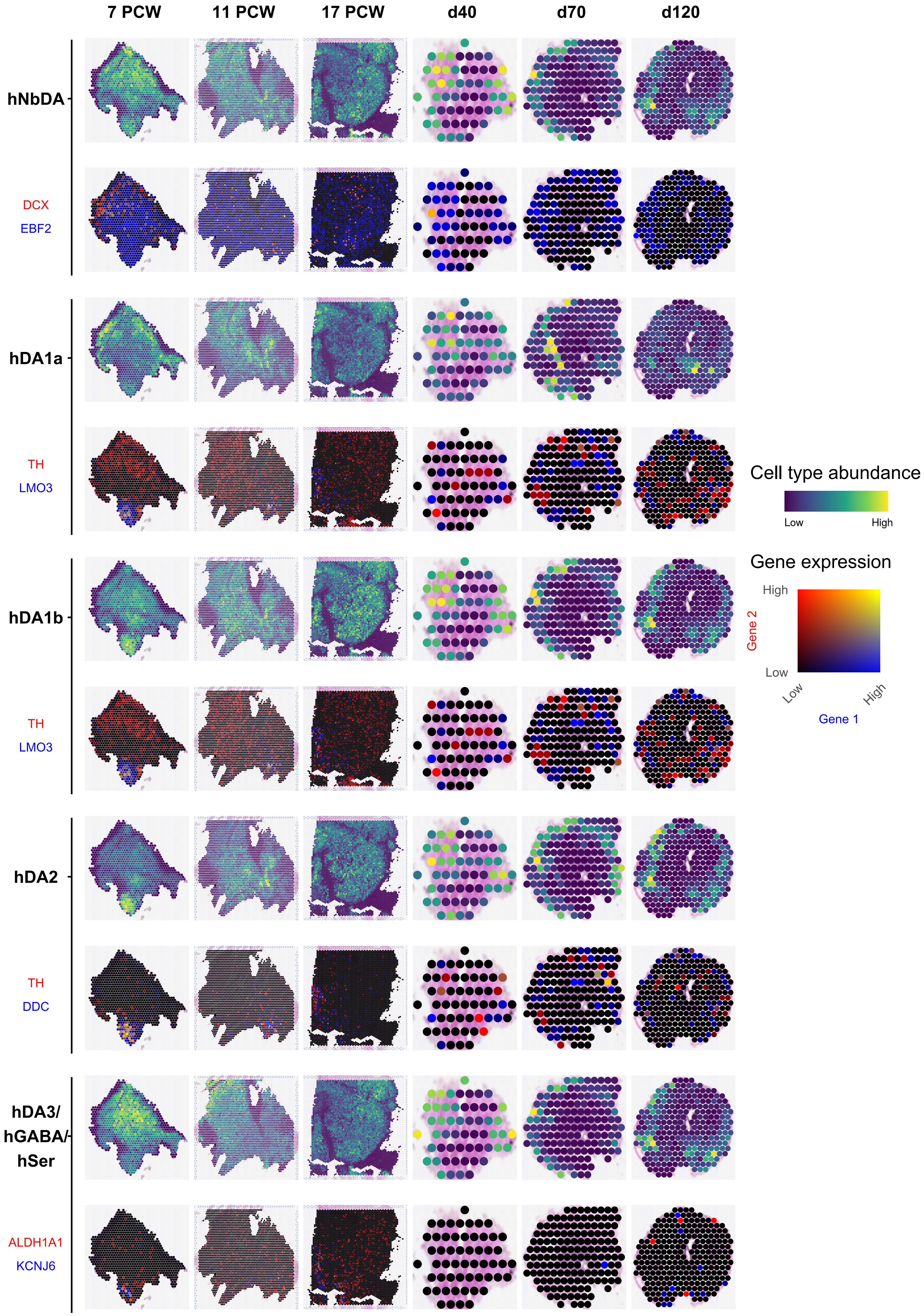

7 PCW
11 PCW
17 PCW
d40
d70
d120
hNbDA
DCX
EBF2
hDA1a
TH
LMO3
hDA1b
TH
LMO3
hDA2
TH
DDC
hDA3/
hGABA/
hSer
ALDH1A1
KCNJ6
Cell type abundance
Low
High
Gene expression
High
Gene 2
Low
Low
High
Gene 1

Supplementary Fig 16 Distribution of dopaminergic populations in fetal tissue and MLOs. Abundance of dopaminergic lineage cells in tissues and organoids, as estimated by the Cell2Location deconvolution algorithm. Expression of two marker genes for each cell type are displayed below.

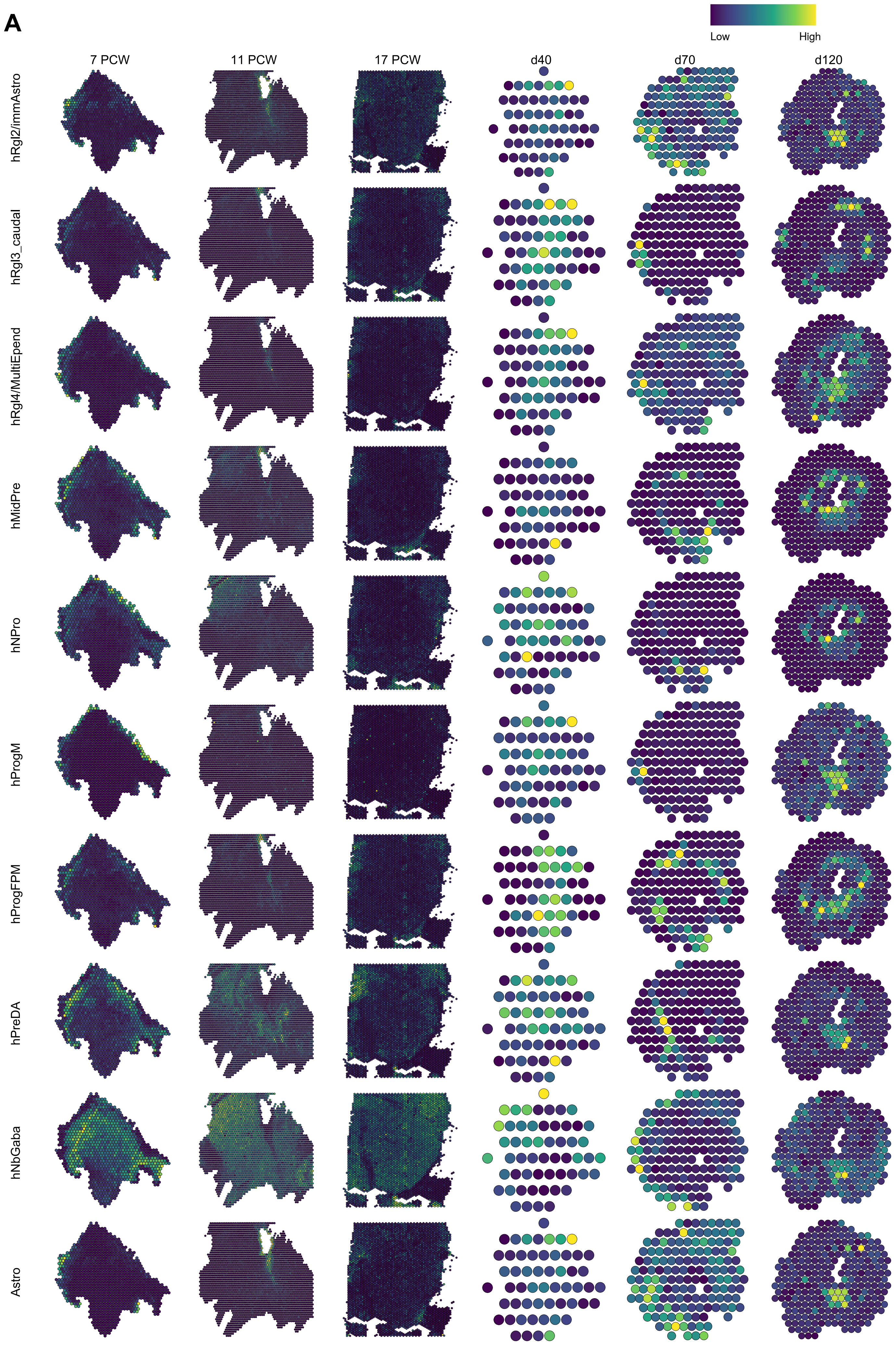
A
Low
High
7 PCW
11 PCW
17 PCW
d40
d70
d120
hRgl2/immAstro
hRgl3_caudal
hRgl4/MultiEpend
hMidPre
hNPro
hProgM
hProgFPM
hPreDA
hNbGaba
Astro

B

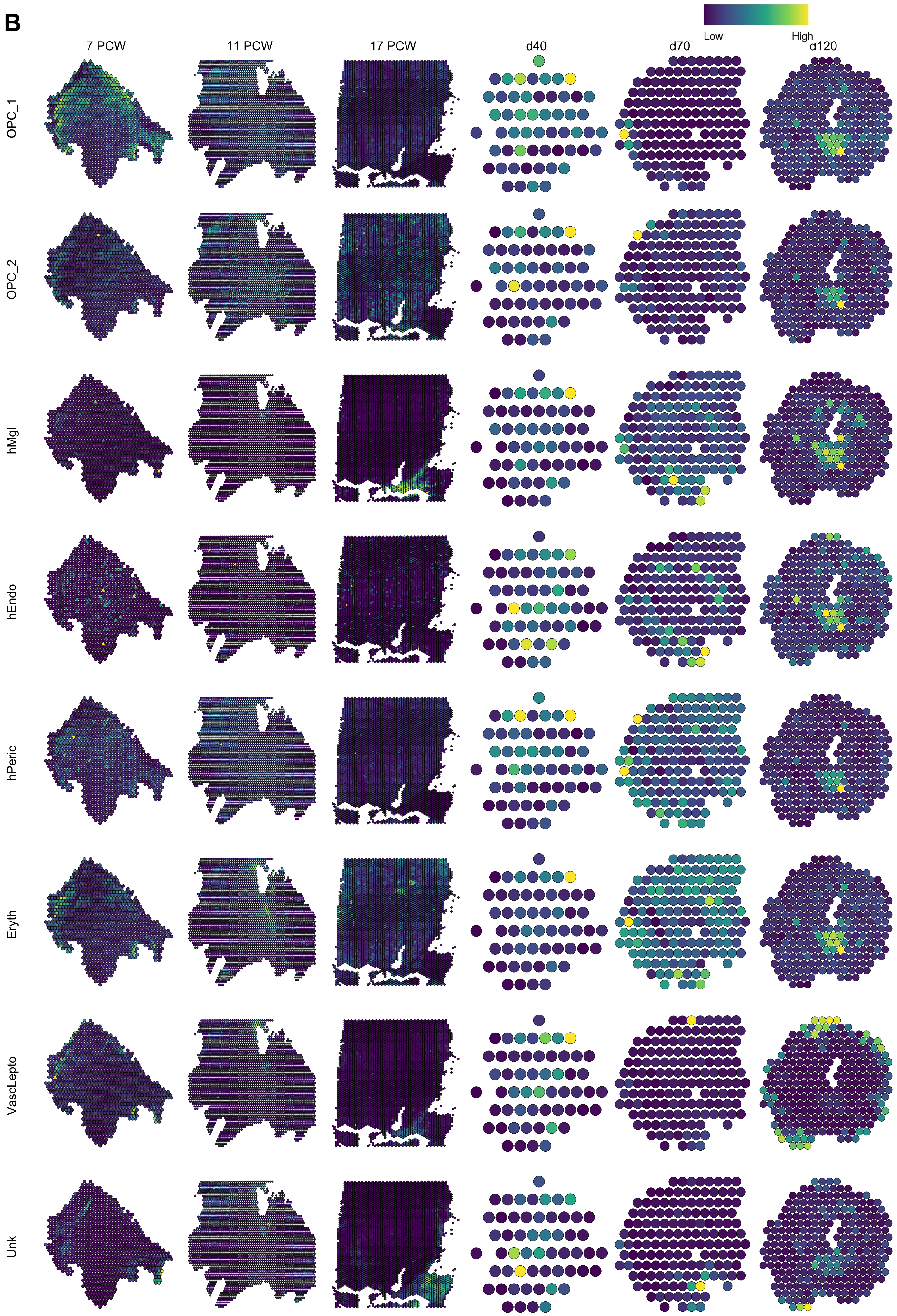
Low
High
7 PCW
11 PCW
17 PCW
d40
d70
d120
OPC_1
OPC_2
hMgl
hEndo
hPeric
Eryth
VascLepto
Unk

Supplementary Fig 17 Distribution of cell types in fetal tissue and MLOs. A,**B**, Abundance of neuronal and non-neuronal cells in tissues (left three columns) and organoids (right three columns), estimated by the Cell2Location deconvolution algorithm.

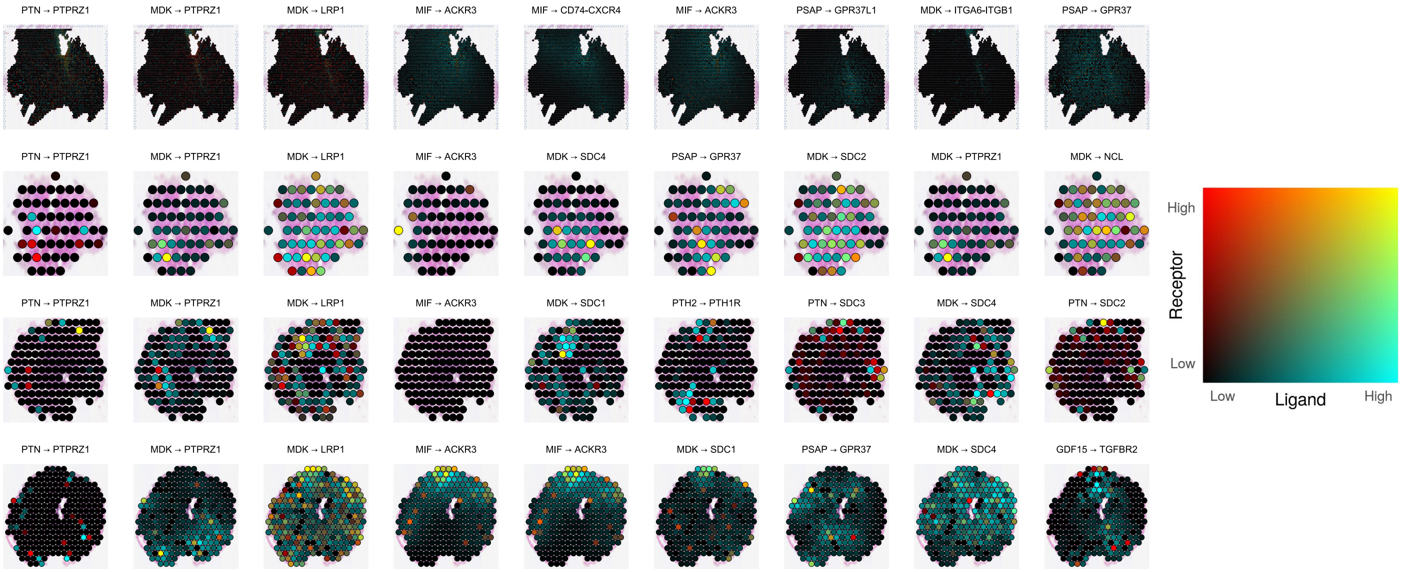
PTN → PTPRZ1
MDK → PTPRZ1
MDK → LRP1
MIF → ACKR3
MIF → CD74-CXCR4
MIF → ACKR3
PSAP → GPR37L1
MDK → ITGA6-ITGB1
PSAP → GPR37
PTN → PTPRZ1
MDK → PTPRZ1
MDK → LRP1
MIF → ACKR3
MDK → SDC4
PSAP → GPR37
MDK → SDC2
MDK → PTPRZ1
MDK → NCL
PTN → PTPRZ1
MDK → PTPRZ1
MDK → LRP1
MIF → ACKR3
MDK → SDC1
PTH2 → PTH1R
PTN → SDC3
MDK → SDC4
PTN → SDC2
PTN → PTPRZ1
MDK → PTPRZ1
MDK → LRP1
MIF → ACKR3
MIF → ACKR3
MDK → SDC1
PSAP → GPR37
MDK → SDC4
GDF15 → TGFBR2
High
Receptor
Low
Low
Ligand
High

Supplementary Fig 18 Distribution of cell-cell communication pathways in fetal tissue and MLOs. A, Expression of pathways at each spot in 11 PCW fetal midbrain and MLOs at 40, 70 and 120 days of differentiation. Computed with COMMOT. Pathways selected according to spatial autocorrelation as determined by the Moran's I non-parametric test.

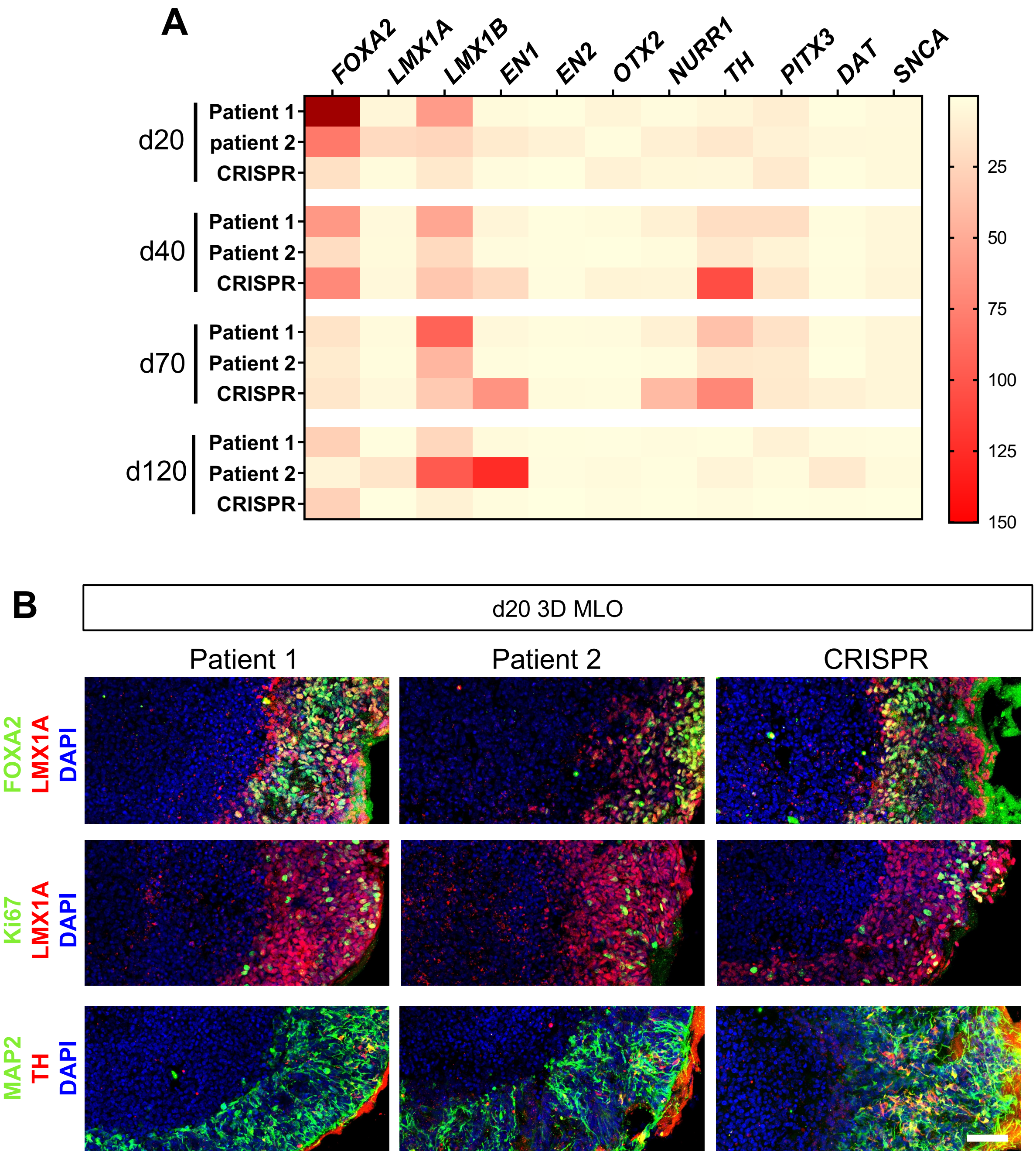

A
FOXA2
LMX1A
LMX1B
EN1
EN2
OTX2
NURR1
TH
PITX3
DAT
SNCA
d20
Patient 1
patient 2
CRISPR
d40
Patient 1
Patient 2
CRISPR
d70
Patient 1
Patient 2
CRISPR
d120
Patient 1
Patient 2
CRISPR
25
50
75
100
125
150
B
d20 3D MLO
Patient 1
Patient 2
CRISPR
FOXA2 LMX1A DAPI
Ki67 LMX1A DAPI
MAP2 TH DAPI

Supplementary Fig 19 Characterization of DTDS derived MLOs. A, qRT-PCR for midbrain related markers *FOXA2*, *LMX1A*, *LMX1B*, *EN1*, *EN2*, *OTX2*, *NURR1*, *PITX3*, *DAT*, *SNCA* relative to housekeeping gene (GAPDH) and normalized to their respective iPSCs (n=3-4 for each line D20-40-70, n=1 for each line D120). **B**, Immunofluorescence analysis for midbrain and neuronal-related proteins FOXA2, LMX1, MAP2 and TH, and Ki67 in Patient 1, Patient 2 and CRISPR corrected isogenic line-derived MLO at 20 days of differentiation. Nuclei are staining with DAPI. Scale bar = 50 µm. Source data are provided as a Source Data file.

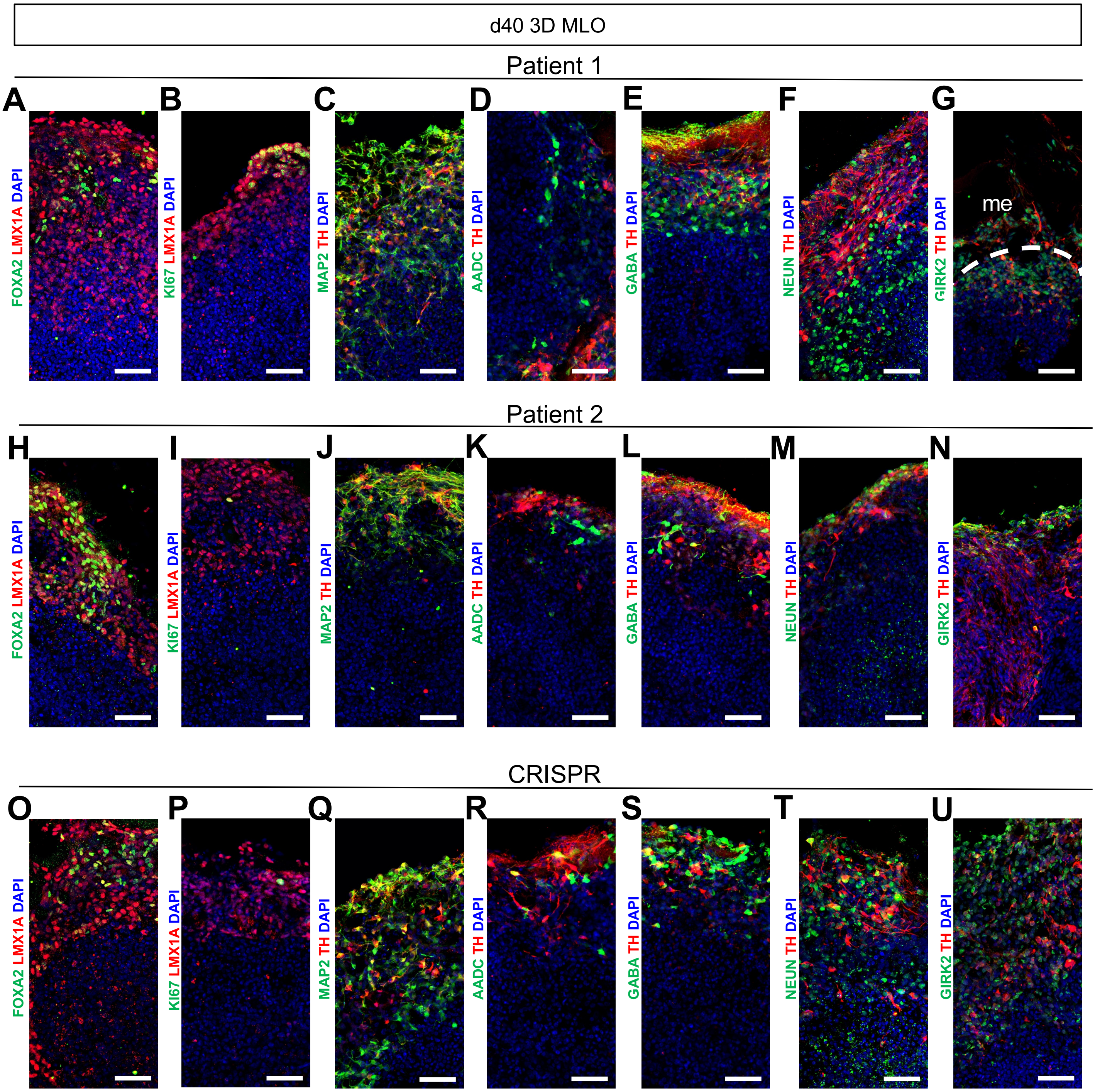
d40 3D MLO
Patient 1
A
FOXA2 LMX1A DAPI
B
KI67 LMX1A DAPI
C
MAP2 TH DAPI
D
AADC TH DAPI
E
GABA TH DAPI
F
NEUN TH DAPI
G
GIRK2 TH DAPI
me
Patient 2
H
FOXA2 LMX1A DAPI
I
KI67 LMX1A DAPI
J
MAP2 TH DAPI
K
AADC TH DAPI
L
GABA TH DAPI
M
NEUN TH DAPI
N
GIRK2 TH DAPI
CRISPR
O
FOXA2 LMX1A DAPI
P
KI67 LMX1A DAPI
Q
MAP2 TH DAPI
R
AADC TH DAPI
S
GABA TH DAPI
T
NEUN TH DAPI
U
GIRK2 TH DAPI

Supplementary Fig 20 Maturation of mDA neurons in DTDS-derived MLOs at 40 days of differentiation. A-U, Representative immunofluorescence images of Patient 1, Patient 2 and CRISPR isogenic lines showing expression of midbrain and neuronal-related proteins FOXA2, LMX1A, MAP2, TH, AADC, GABA, NEUN and GIRK2 and the proliferative marker Ki67. Nuclei are staining with DAPI. Scale bars = 50 μm. Matrigel embedding (me).

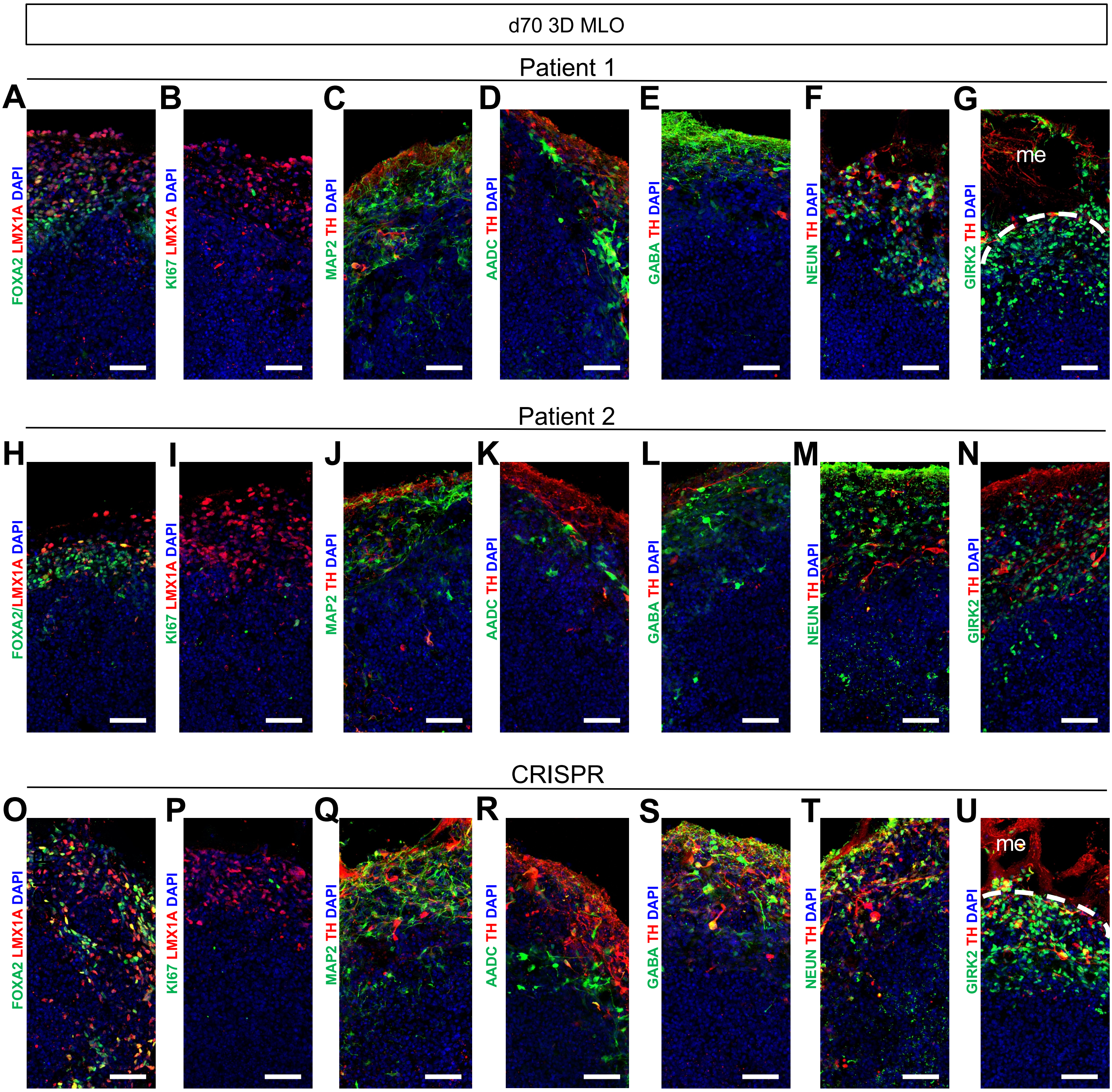
d70 3D MLO
Patient 1
A
B
C
D
E
F
G
FOXA2 LMX1A DAPI
KI67 LMX1A DAPI
MAP2 TH DAPI
AADC TH DAPI
GABA TH DAPI
NEUN TH DAPI
GIRK2 TH DAPI
me
Patient 2
H
I
J
K
L
M
N
FOXA2/LMX1A DAPI
KI67 LMX1A DAPI
MAP2 TH DAPI
AADC TH DAPI
GABA TH DAPI
NEUN TH DAPI
GIRK2 TH DAPI
CRISPR
O
P
Q
R
S
T
U
FOXA2 LMX1A DAPI
KI67 LMX1A DAPI
MAP2 TH DAPI
AADC TH DAPI
GABA TH DAPI
NEUN TH DAPI
GIRK2 TH DAPI
me

Supplementary Fig 21 Maturation of mDA neurons in DTDS-derived MLOs at 70 days of differentiation. A-U, Representative immunofluorescence images of Patient 1, Patient 2 and CRISPR isogenic lines showing expression of midbrain and neuronal-related proteins FOXA2, LMX1A, MAP2, TH, AADC, GABA, NEUN and GIRK2 and the proliferative marker Ki67. Nuclei are staining with DAPI. Scale bars = 50 μm. Matrigel embedding (me).

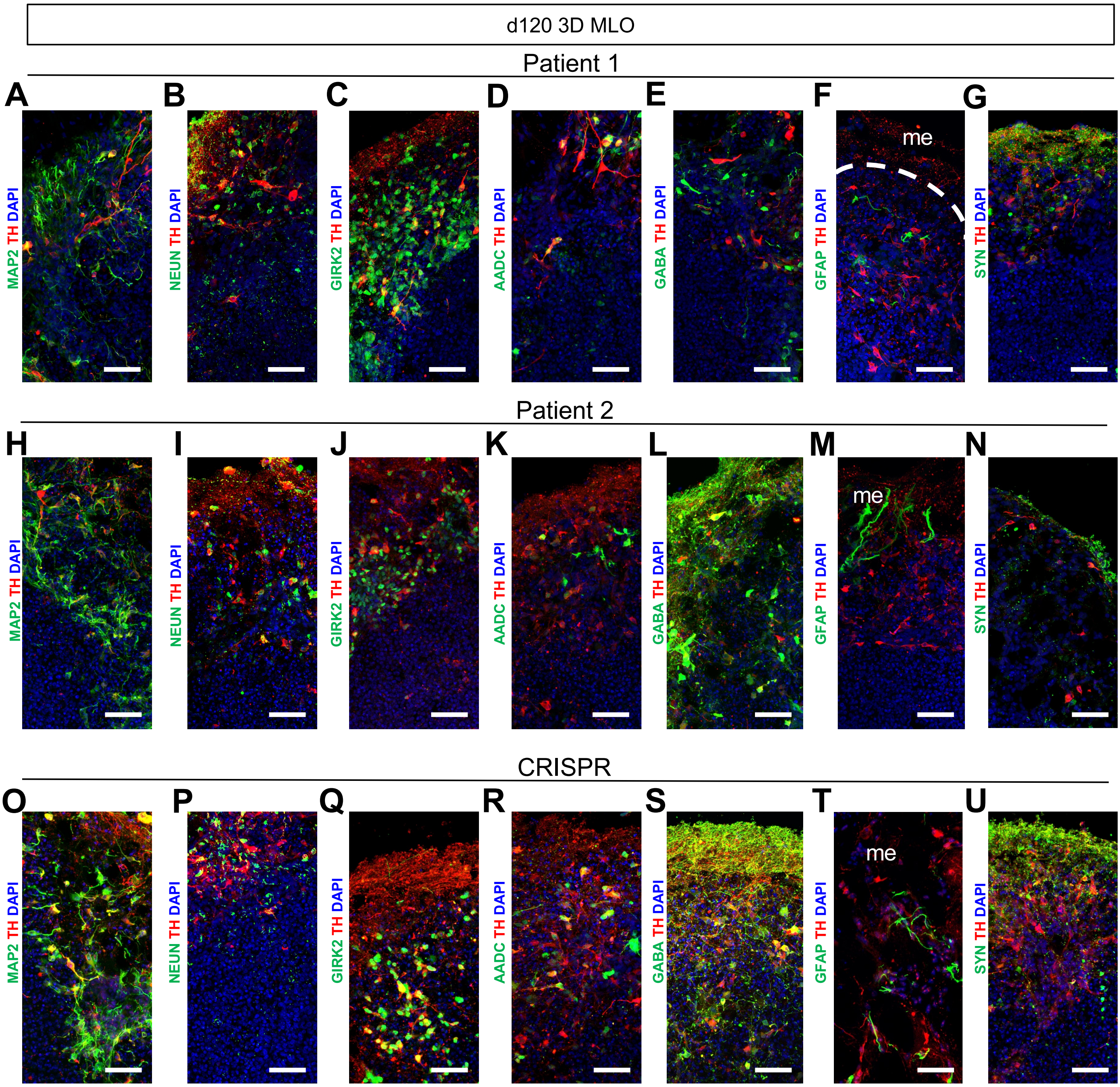
d120 3D MLO
Patient 1
A
MAP2 TH DAPI
B
NEUN TH DAPI
C
GIRK2 TH DAPI
D
AADC TH DAPI
E
GABA TH DAPI
F
GFAP TH DAPI
me
G
SYN TH DAPI
Patient 2
H
MAP2 TH DAPI
I
NEUN TH DAPI
J
GIRK2 TH DAPI
K
AADC TH DAPI
L
GABA TH DAPI
M
GFAP TH DAPI
me
N
SYN TH DAPI
CRISPR
O
MAP2 TH DAPI
P
NEUN TH DAPI
Q
GIRK2 TH DAPI
R
AADC TH DAPI
S
GABA TH DAPI
T
GFAP TH DAPI
me
U
SYN TH DAPI

Supplementary Fig 22 Maturation of mDA neurons in DTDS-derived MLOs at 70 days of differentiation. A-U, Representative immunofluorescence images of Patient 1, Patient 2 and CRISPR isogenic lines showing expression of mDA neuron-related proteins TH, AADC and GIRK2, mature neuronal makers MAP2, NEUN, SYP, neural cells GABA positive cells and GFAP positive glial cells. Nuclei are staining with DAPI. Scale bars = 50 μm. Necrotic core (nc), Matrigel embedding (me).

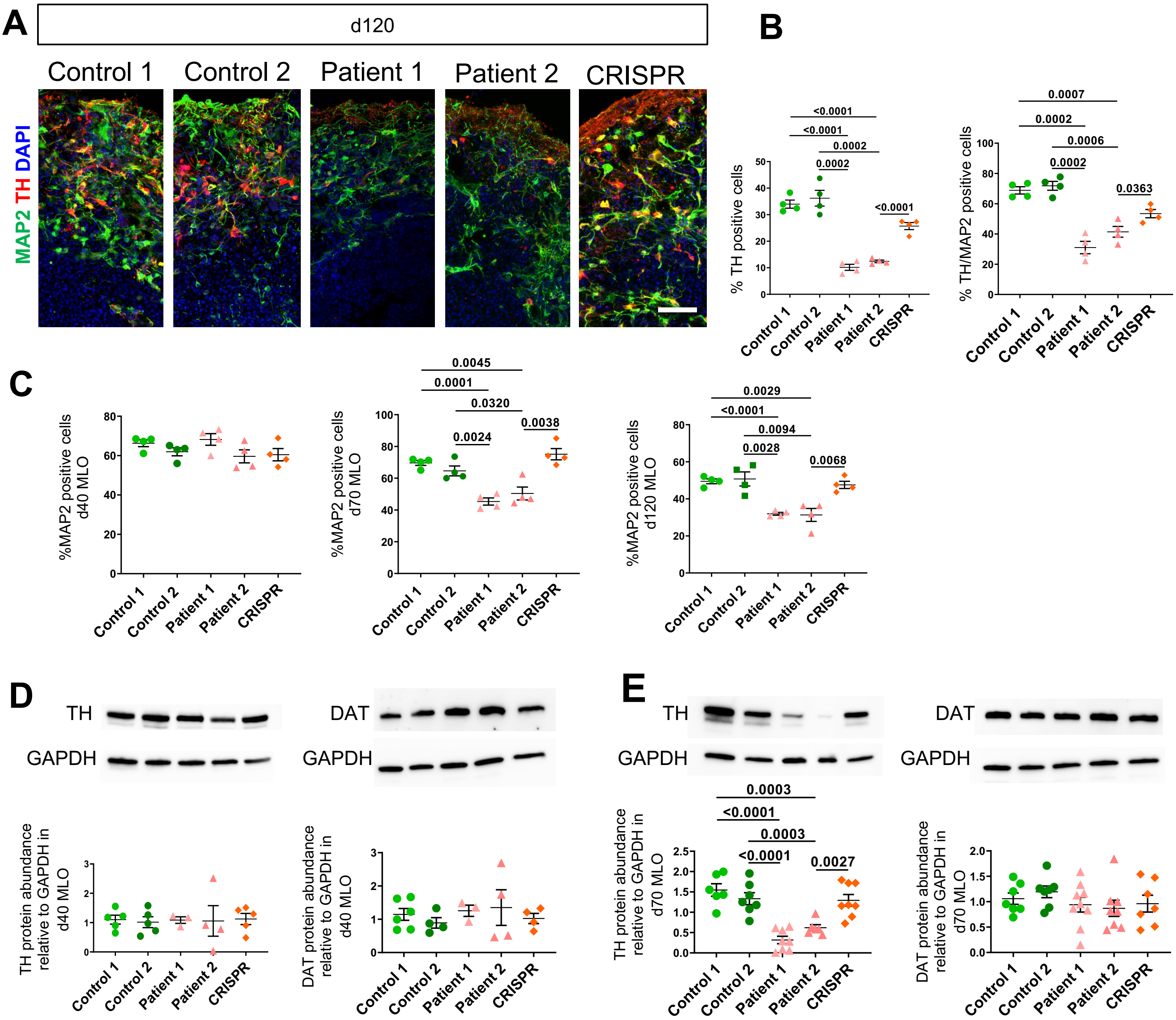
A
d120
Control 1
Control 2
Patient 1
Patient 2
CRISPR
MAP2 TH DAPI
B
% TH positive cells
<0.0001
<0.0001
0.0002
0.0002
<0.0001
% TH/MAP2 positive cells
0.0007
0.0002
0.0006
0.0002
0.0363
C
%MAP2 positive cells d40 MLO
%MAP2 positive cells d70 MLO
0.0045
0.0001
0.0320
0.0024
0.0038
%MAP2 positive cells d120 MLO
0.0029
<0.0001
0.0094
0.0028
0.0068
D
TH
GAPDH
DAT
GAPDH
TH protein abundance relative to GAPDH in d40 MLO
DAT protein abundance relative to GAPDH in d40 MLO
E
TH
GAPDH
DAT
GAPDH
TH protein abundance relative to GAPDH in d70 MLO
0.0003
<0.0001
0.0003
<0.0001
0.0027
DAT protein abundance relative to GAPDH in d70 MLO

Supplementary Fig 23 DTDS MLOs show loss of mDA neurons and reduced TH protein content. **A**, Immunofluorescence analysis of control and DTDS patient-derived MLOs for TH and MAP2 at 120 days of differentiation. Nuclei are staining with DAPI. Scale bar = 50 μm. **B**, Quantification of TH positive and TH/MAP2 double positive neurons for patients and controls lines at 120 days of differentiation. **C**, Quantification of MAP2 total positive cells in DTDS control and patient-derived MLOs at 40, 70 and 120 days of differentiation. **D-E**, Western blot analysis for TH and DAT and relative protein abundance to GAPDH in DTDS control and patient-derived MLOs at 40 and 70 days of differentiation respectively. Error bars indicate SEM. DTDS lines were independently compared to controls using two-tailed Student's *t*-test for all analyses. Statistically significant differences indicate comparison of Control 1 or 2 to Patient 1 or 2 and Patient 2 to CRISPR. Source data are provided as a Source Data file.

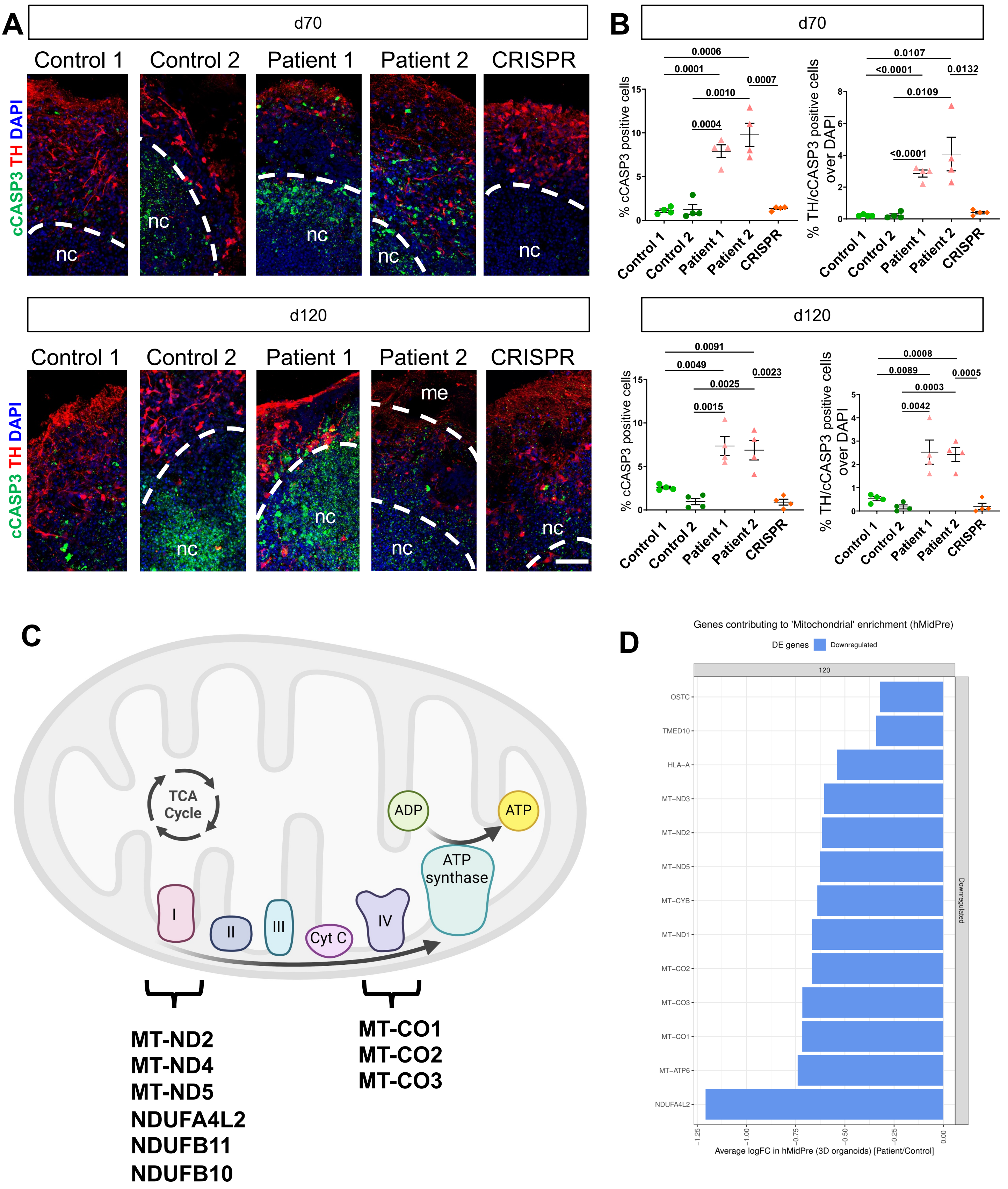

A
d70
Control 1
Control 2
Patient 1
Patient 2
CRISPR
cCASP3 TH DAPI
nc
d120
me
B
% cCASP3 positive cells
% TH/cCASP3 positive cells over DAPI
0.0006
0.0001
0.0010
0.0007
0.0004
0.0107
<0.0001
0.0132
0.0109
<0.0001
0.0091
0.0049
0.0025
0.0023
0.0015
0.0008
0.0089
0.0005
0.0003
0.0042
C
TCA Cycle
ADP
ATP
ATP synthase
I
II
III
Cyt C
IV
MT-ND2
MT-ND4
MT-ND5
NDUFA4L2
NDUFB11
NDUFB10
MT-CO1
MT-CO2
MT-CO3
D
Genes contributing to 'Mitochondrial' enrichment (hMidPre)
DE genes
Downregulated
120
OSTC
TMED10
HLA-A
MT-ND3
MT-ND2
MT-ND5
MT-CYB
MT-ND1
MT-CO2
MT-CO3
MT-CO1
MT-ATP6
NDUFA4L2
Average logFC in hMidPre (3D organoids) [Patient/Control]

Supplementary Fig 24 DTDS MLOs show increased levels of cCASP3 positive cells. A, Immunofluorescence analysis of control and DTDS patient-derived MLOs for cleaved CASP3 (cCASP3) and TH at 40 and 70 days of differentiation. Nuclei are staining with DAPI. Positive cells located in the necrotic core (nc) were excluded from the quantification. Scale bar = 50 µm. **B**, Quantification of total cCASP3 positive cells and TH/ cCASP3. Error bars indicate SEM. DTDS lines were independently compared to controls using two-tailed Student's *t*-test for all analyses. Statistically significant differences indicate comparison of Control 1 or 2 to Patient 1 or 2 and Patient 2 to CRISPR. Necrotic core (nc). **C**, Graphic representation of mitochondria genes dysregulated in DTDS mDA neurons and role in mitochondrial respiratory chain. Created in BioRender. BUDINGER, D. (2025) https://BioRender.com/gddkq14. **D**, Average LogFC for mitochondrial genes downregulated in patients MLOs compared to controls for midbrain precursors cell type (hMidPre). Matrigel embedding (me). Source data are provided as a Source Data file.

Uncropped western blot scans in supplementary figure 23

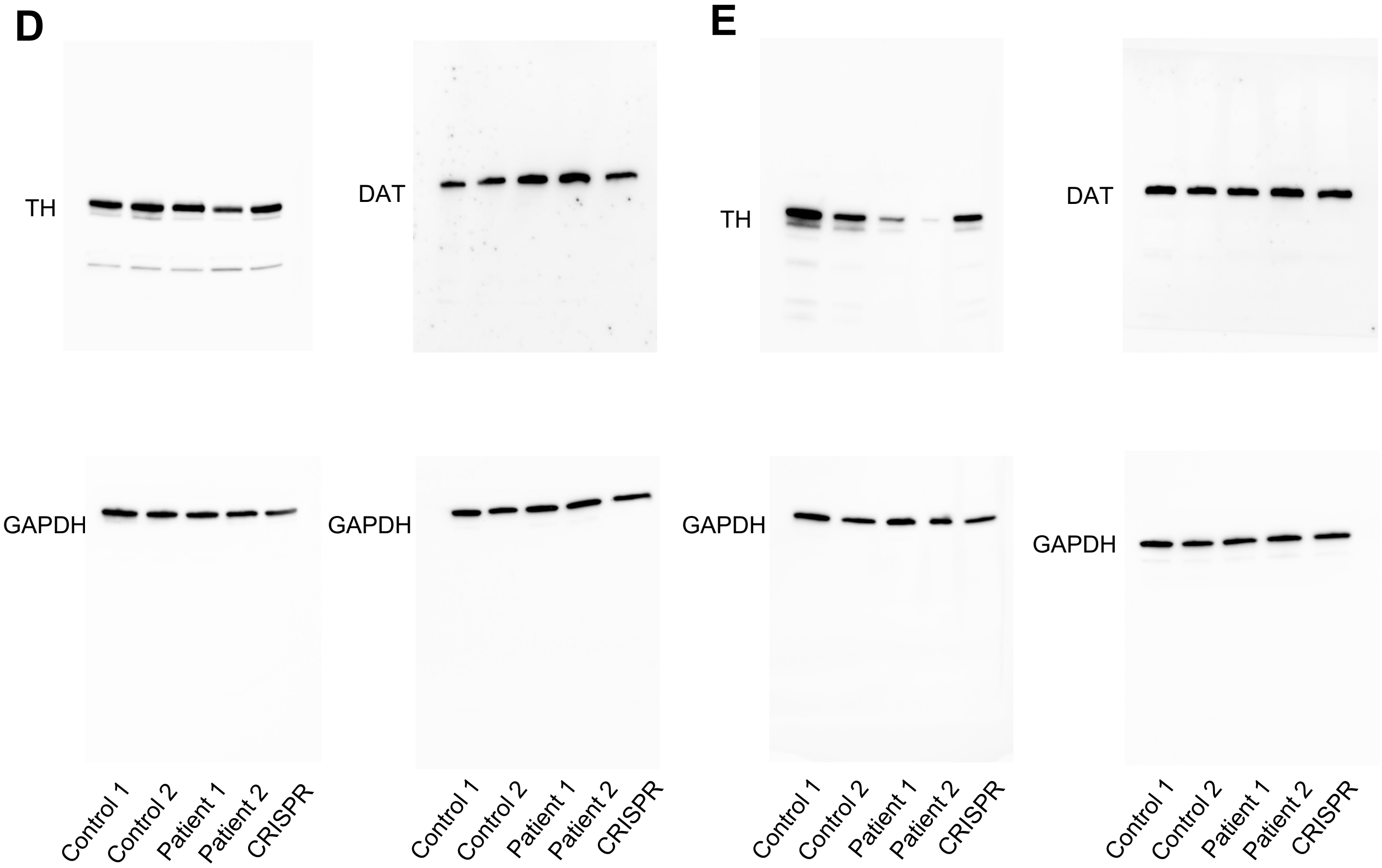

Supplement: Supplementary file 1 — Supplementary Figs. [file 41467_2025_67779_MOESM1_ESM.pdf]
