# Supplementary material for: An in vivo and in vitro spatiotemporal profile of human midbrain development
Source: Nat Commun. 2026 Feb 3;17:1354. doi: 10.1038/s41467-025-67779-1 (PMC12877092; doi:10.1038/s41467-025-67779-1)
Supplement: Supplementary file 2 — Description of Additional Supplementary Files [file 41467_2025_67779_MOESM2_ESM.pdf]

An in vivo and in vitro spatiotemporal profile of human midbrain development

Description of Additional Supplementary Files 1-16

File name: Supplementary Data S1

Description: List of fetal collected samples with their relative age and data profiling modalities (immunohistochemistry, scRNA-seq or Spatial transcriptomics).

File name: Supplementary Data S2

Description: 10x libraries or sample ("midBrainId") that were pre-processed with CellRanger.

File name: Supplementary Data S3

Description: Marker genes used for cell type annotation in the scRNA-seq dataset

File name: Supplementary Data S4

Description: Gene ontology enrichment analysis of the differentially expressed (DE) genes between 3D and 2D in vitro models within dopaminergic populations (hDA1a, hDA1b, hDA2). DE genes (upregulated) obtained from testDiffExp() from miloR function and GO enrichment analysed with gprofiler2 R package. GO enrichment test: hypergeometric test for over-representation analysis implemented in gprofiler2 R package (gost).

File name: Supplementary Data S5

Description: Gene ontology enrichment analysis of the differentially expressed (DE) genes between fetal and 3D in vitro model (hDA2 population). DE genes computed with FindMarkers() function from Seurat and GO enrichment with GOstats R package. GO enrichment test: hypergeometric test for over-representation analysis implemented in gprofiler2 R package (gost).

File name: Supplementary Data S6

Description: Cross-referencing of unified and dataset-specific cell type annotation.

File name: Supplementary Data S7

Description: Pseudotime inference and corresponding ranked cell order comparison across Slingshot, Destiny and Monocle3.

File name: Supplementary Data S8

Description: Modules of co-regulated genes that change as a function of pseudotime. For each gene, a module and a supermodule are indicated, jointly with the gene embeddings for the UMAP.MNN projection.

File name: Supplementary Data S9

Description: Visium Platform loadings.

File name: Supplementary Data S10

Description: Differentially expressed genes along maturation paths inferred from spatial transcriptomics, using C-SIDE differential expression detection package.

File name: Supplementary Data S11

Description: Differentially expressed genes (upregulated and downregulated) between patients and controls in 3D in vitro model. All cell types (with at least 10

cells for both experimental conditions) were tested across differentiation days 40, 70 and 120. GO enrichment test: hypergeometric test for over-representation analysis implemented in gprofiler2 R package (gost).

File name: Supplementary Data S12

Description: List of primary antibodies used.

File name: Supplementary Data S13

Description: Target mix for genes (primer sequences).

File name: Supplementary Data S14

Description: Donor lines used in each of the pooled 10x libraries (midBrainId refers to the 10x library, or processed GEM well).

File name: Supplementary Data S15

Description: Genes with significant expression changes between the start and the end of the dopaminergic neurons trajectory (our dataset) as detected by tradeSeq (startVsEndTest, adjusted $p<0.001$, abs(logFC)>2).

File name: Supplementary Data S16

Description: Genes with significant expression changes between the start and the end of the astrocyte trajectory (our dataset) as detected by tradeSeq (startVsEndTest, adjusted $p<0.001$, abs(logFC)>2).
