# Supplementary material for: An in vivo and in vitro spatiotemporal profile of human midbrain development
Source: Nat Commun. 2026 Feb 3;17:1354. doi: 10.1038/s41467-025-67779-1 (PMC12877092; doi:10.1038/s41467-025-67779-1)
Supplement: Supplementary file 4 — Reporting Summary [file 41467_2025_67779_MOESM4_ESM.pdf]

nature portfolio

Corresponding author(s): Serena Barral

Last updated by author(s): 12 November 2025

Reporting Summary

Nature Portfolio wishes to improve the reproducibility of the work that we publish. This form provides structure for consistency and transparency in reporting. For further information on Nature Portfolio policies, see our Editorial Policies and the Editorial Policy Checklist.

Please do not complete any field with "not applicable" or n/a. Refer to the help text for what text to use if an item is not relevant to your study.
For final submission: please carefully check your responses for accuracy; you will not be able to make changes later.

Statistics

For all statistical analyses, confirm that the following items are present in the figure legend, table legend, main text, or Methods section.

n/a	Confirmed	
☐	☑	The exact sample size (*n*) for each experimental group/condition, given as a discrete number and unit of measurement
☐	☑	A statement on whether measurements were taken from distinct samples or whether the same sample was measured repeatedly
☐	☑	The statistical test(s) used AND whether they are one- or two-sided<br>*Only common tests should be described solely by name; describe more complex techniques in the Methods section.*
☐	☑	A description of all covariates tested
☐	☑	A description of any assumptions or corrections, such as tests of normality and adjustment for multiple comparisons
☐	☑	A full description of the statistical parameters including central tendency (e.g. means) or other basic estimates (e.g. regression coefficient) AND variation (e.g. standard deviation) or associated estimates of uncertainty (e.g. confidence intervals)
☐	☑	For null hypothesis testing, the test statistic (e.g. *F*, *t*, *r*) with confidence intervals, effect sizes, degrees of freedom and *P* value noted<br>*Give P values as exact values whenever suitable.*
☒	☐	For Bayesian analysis, information on the choice of priors and Markov chain Monte Carlo settings
☒	☐	For hierarchical and complex designs, identification of the appropriate level for tests and full reporting of outcomes
☐	☑	Estimates of effect sizes (e.g. Cohen's *d*, Pearson's *r*), indicating how they were calculated

Our web collection on statistics for biologists contains articles on many of the points above.

Software and code

Policy information about availability of computer code

Data collection: Described in "Methods".

Data analysis: Two GitHub repositories are available for reproducing the analysis and figures of the manuscript: one for single-cell transcriptomics (https://github.com/paupuigdevall/MLOscRNAseq, archived at https://doi.org/10.5281/zenodo.17541380) and another for spatial transcriptomics (https://github.com/george-hall-ucl/mlo_spatial_transcriptomics archived at https://doi.org/10.5281/zenodo.17550243). These repositories include scripts for processing steps, data analysis and figure generation. Additionally, the Docker images used for spatial transcriptomics are is available on Dockerhub (main pipeline: georgehallucl/midbrain_tissues_organoids_docker; commot: georgehallucl/commot_docker; cell2location: georgehallucl/cell2location_docker). An archive to reproduce the spatial transcriptomics analysis is available at https://figshare.com/s/01ec7b05ef9f28e000ca.

For single-cell transcriptomics:
CellRanger (version 3.1.0)
Harmony (v.0.1.0)
Seurat R package (v.4.1.3)
miloR package (v.1.2.0)
Scanpy workflow (version 1.10.4)
Seurat package (v.5.0.0)
SeuratWrappers, v.0.3.19
ComplexHeatmap R package (v2.18.0)
Slingshot (v.2.10.0)
Destiny (v.3.16.0)
Monocle3 (v.1.2.9)

For spatial transcriptomics
spaceranger (v2.1.0)
Seurat (v4.4.0)
Cell2Location (v0.1.3)
spacexr package v2.2.1
COMMOT (v0.0.3)
CellChat database (v1)

For manuscripts utilizing custom algorithms or software that are central to the research but not yet described in published literature, software must be made available to editors and reviewers. We strongly encourage code deposition in a community repository (e.g. GitHub). See the Nature Portfolio guidelines for submitting code & software for further information.

Data

Policy information about availability of data

All manuscripts must include a data availability statement. This statement should provide the following information, where applicable:

- Accession codes, unique identifiers, or web links for publicly available datasets
- A description of any restrictions on data availability
- For clinical datasets or third party data, please ensure that the statement adheres to our policy

Raw FASTQ files for both single-cell and spatial transcriptomics generated in this study have been deposited in Gene Expression Omnibus (GEO) under assession code (GEO accession GSE277032). The four supplemental data files generated in this study are available in a Zenodo repository (https://zenodo.org/records/13765496), detailed as follows: Data S1-S2: VCF files with donor genotypes to deconvolute pooled scRNA-seq data. Data S3: Single-cell data generated in our study (RDS object: Seurat object with counts and metadata). Data S4: Integrated datasets (RDS object: Seurat object with counts and metadata). Additionally, the images from the immunohistochemistry analysis on midbrain tissues are available in the MRC-Wellcome Trust Human Developmental Biology Resource (HDBR) Atlas (https://hdbratlas.org/gene-expression/midbrain.html).

Research involving human participants, their data, or biological material

Policy information about studies with human participants or human data. See also policy information about sex, gender (identity/presentation), and sexual orientation and race, ethnicity and racism.

Reporting on sex and gender	Controls and patients are both composed of one female and one male.
Reporting on race, ethnicity, or other socially relevant groupings	Not reported.
Population characteristics	Not reported.
Recruitment	Not reported.
Ethics oversight	The UK NHS Health Research Authority, London Hampstead Research Ethics Committee has approved the work with induced pluripotent stem cells under the REC reference number 13/LO/0171, IRAS project ID: 100318. Fetal samples have been collected under the ethical approval REC reference number 23/NE/0135, IRAS project ID: 330783 and REC reference number 23/LO/0312, IRAS project ID: 326492, granted to Human Developmental Biology Resource (HDBR) by UK NHS Health Research Authority, North East - Newcastle & North Tyneside 1 Research Ethics Committee.

Note that full information on the approval of the study protocol must also be provided in the manuscript.

Field-specific reporting

Please select the one below that is the best fit for your research. If you are not sure, read the appropriate sections before making your selection.

☑ Life sciences ☐ Behavioural & social sciences ☐ Ecological, evolutionary & environmental sciences

For a reference copy of the document with all sections, see nature.com/documents/nr-reporting-summary-flat.pdf

Life sciences study design

All studies must disclose on these points even when the disclosure is negative.

Sample size	All key experiments were independently replicated to ensure the robustness and reproducibility of findings. For each assay type, independent biological replicates were performed using samples from distinct tissue donors and/or model systems. Spatial transcriptomics experiments were conducted using tissue from four independent donors and six model samples (derived from two donors across three timepoints). Single-cell RNA sequencing experiments were performed using four tissue donors and fifteen model samples (five donors across three timepoints). Molecular analyses were repeated using samples from five tissue donors and five model donors, with replicate numbers detailed in the figure legends. All attempts at replication produced consistent results across donors and timepoints, confirming the reproducibility of the main findings. Sample sizes were determined based on standard practice in the field and prior experience with similar transcriptomic and molecular assays, ensuring sufficient power to detect biologically meaningful differences across donors and experimental timepoints. No formal statistical power calculation was performed. Instead, sample sizes were chosen to balance experimental feasibility, donor availability, and statistical reliability. The selected numbers of biological replicates (n=4–5 donors per group, with multiple timepoints for model systems) were sufficient to achieve consistent and statistically robust results, as reflected by the reproducibility and significance of the reported data (PMID:37443770, PMID:34911939, PMID:27716510, PMID:40369074).
Data exclusions	Some ST samples excluded due to tearing. Western blot: excluded both highest and lowest outliers in some cases.
Replication	In ST and single-cell models, 2 independent differentiations. Molecular analysis - immunofluorescence: 4 replications each; Western blot: specified in image.
Randomization	The in vitro scRNA-seq samples profiled in the same time point were merged in three different pools previous to sequencing following a sample randomisation to minimize batch effects. Similarly, foetal samples were sequenced in two different batches merging at least two samples with different developmental stage.
Blinding	NA as not a clinical trial.

Behavioural & social sciences study design

All studies must disclose on these points even when the disclosure is negative.

Study description	
Research sample	
Sampling strategy	
Data collection	
Timing	
Data exclusions	
Non-participation	
Randomization	

nature portfolio | reporting summary

Ecological, evolutionary & environmental sciences study design

All studies must disclose on these points even when the disclosure is negative.

Study description	
Research sample	
Sampling strategy	
Data collection	
Timing and spatial scale	
Data exclusions	
Reproducibility	
Randomization	
Blinding	

Did the study involve field work? ☐ Yes ☐ No

Field work, collection and transport

Field conditions	
Location	
Access & import/export	
Disturbance	

Reporting for specific materials, systems and methods

We require information from authors about some types of materials, experimental systems and methods used in many studies. Here, indicate whether each material, system or method listed is relevant to your study. If you are not sure if a list item applies to your research, read the appropriate section before selecting a response.

Materials & experimental systems

n/a	Involved in the study
☐	☑ Antibodies
☐	☑ Eukaryotic cell lines
☒	☐ Palaeontology and archaeology
☒	☐ Animals and other organisms
☒	☐ Clinical data
☒	☐ Dual use research of concern
☒	☐ Plants

Methods

n/a	Involved in the study
☒	☐ ChIP-seq
☒	☐ Flow cytometry
☒	☐ MRI-based neuroimaging

Antibodies

Antibodies used

Name	Use, concentration	Source, catalogue number
AADC	IF, 1:5000	Thermo Scientific, PA5-25450
ALDH1A1	IF, 1:250	Abcam, ab52492
CALB1	IF, 1:500	Sigma-Aldrich C9848
Cleaved Caspase-3	IF, 1:400	Cell Signaling Tech., Asp175
CORIN	IF,	R&D Systems MAB2209
DAT	IF, 1:100	Millipore, MAB369
DAT	WB, 1:3000	Millipore, AB1766
FOXA2	IF, 1:500	BD Pharmigen, 561580
FOXA2	IF, 1:50	ThermoFisher Scientific, TA500073
GABA	IF, 1:1000	Sigma A2052
GAPDH-HRP	WB, 1:3000	Cell Signaling Tech, 3683
GFAP	IF, 1:400	Millipore, MAB3402
GIRK2	IF, 1:400	Alomone Labs, APC-006
Ki67	IF, 1:100	BD Pharmingen 550609
LMX-1	IF, 1:2000	Millipore, AB10533
LMX1A	IF, 1:750	Abcam, ab139726
MAP2	IF, 1:400	Sigma-Aldrich, M9942
NeuN	IF, 1:100	R&D Systems MAB377
OTX2	IF,1:500	R&D Systems AF1979
PITX3	IF, 1:200	Invitrogen, 701181
SOX2	IF, 1:200	Cell Signaling Technology, D6D9
SOX2	IF, 1:100	R&D Systems MAB2018
Synaptophisin	IF, 1:400	Sigma-Aldrich, SAB4502906
TH	IF, 1:400	Aves Labs, TYH
TH	WB, 1:3000	Millipore, AB152

Validation

Eukaryotic cell lines

Policy information about cell lines and Sex and Gender in Research

Cell line source(s)	two patient lines with missense mutations in SLC6A3 (patient 1: c.1103T>A, p.L368Q; patient 2: c.1184C>T, p.P395L) and a CRISPR-Cas9 corrected isogenic line from patient 2 (Ng et al. 2021).<br>Control 1: Male<br>Control 2: Female<br>Patient 1: Male<br>Patient 2: Female<br>Isogenic control patient 2: Female
Authentication	Provided in "Materials and Methods".
Mycoplasma contamination	Free from mycoplasma contamination.
Commonly misidentified lines (See ICLAC register)	Not present.

Palaeontology and Archaeology

Specimen provenance	
Specimen deposition	
Dating methods	

☐ Tick this box to confirm that the raw and calibrated dates are available in the paper or in Supplementary Information.

Ethics oversight	

Note that full information on the approval of the study protocol must also be provided in the manuscript.

Animals and other research organisms

Policy information about studies involving animals; ARRIVE guidelines recommended for reporting animal research, and Sex and Gender in Research

Laboratory animals	
Wild animals	
Reporting on sex	
Field-collected samples	
Ethics oversight	

Note that full information on the approval of the study protocol must also be provided in the manuscript.

Clinical data

Policy information about clinical studies

All manuscripts should comply with the ICMJE guidelines for publication of clinical research and a completed CONSORT checklist must be included with all submissions.

Clinical trial registration	
Study protocol	
Data collection	
Outcomes	

Dual use research of concern

Policy information about dual use research of concern

Hazards

Could the accidental, deliberate or reckless misuse of agents or technologies generated in the work, or the application of information presented in the manuscript, pose a threat to:

No	Yes	
☐	☐	Public health
☐	☐	National security
☐	☐	Crops and/or livestock
☐	☐	Ecosystems
☐	☐	Any other significant area

Experiments of concern

Does the work involve any of these experiments of concern:

No	Yes	
☐	☐	Demonstrate how to render a vaccine ineffective
☐	☐	Confer resistance to therapeutically useful antibiotics or antiviral agents
☐	☐	Enhance the virulence of a pathogen or render a nonpathogen virulent
☐	☐	Increase transmissibility of a pathogen
☐	☐	Alter the host range of a pathogen
☐	☐	Enable evasion of diagnostic/detection modalities
☐	☐	Enable the weaponization of a biological agent or toxin
☐	☐	Any other potentially harmful combination of experiments and agents

Plants

Seed stocks

Novel plant genotypes

Authentication

ChIP-seq

Data deposition

☐ Confirm that both raw and final processed data have been deposited in a public database such as GEO.

☐ Confirm that you have deposited or provided access to graph files (e.g. BED files) for the called peaks.

Data access links
May remain private before publication.

Files in database submission

Genome browser session
(e.g. UCSC)

Methodology

Replicates

Sequencing depth

Antibodies

Peak calling parameters

Data quality

Software

Flow Cytometry

Plots

Confirm that:

- [ ] The axis labels state the marker and fluorochrome used (e.g. CD4-FITC).
- [ ] The axis scales are clearly visible. Include numbers along axes only for bottom left plot of group (a 'group' is an analysis of identical markers).
- [ ] All plots are contour plots with outliers or pseudocolor plots.
- [ ] A numerical value for number of cells or percentage (with statistics) is provided.

Methodology

Sample preparation

Instrument

Software

Cell population abundance

Gating strategy

- [ ] Tick this box to confirm that a figure exemplifying the gating strategy is provided in the Supplementary Information.

Magnetic resonance imaging

Experimental design

Design type

Design specifications

Behavioral performance measures

Imaging type(s)

Field strength

Sequence & imaging parameters

Area of acquisition

Diffusion MRI ☐ Used ☐ Not used

Preprocessing

Preprocessing software

Normalization

Normalization template

Noise and artifact removal

Volume censoring

Statistical modeling & inference

Model type and settings

Effect(s) tested

Specify type of analysis: ☐ Whole brain ☐ ROI-based ☐ Both

Statistic type for inference

(See Eklund et al. 2016)

Correction

Models & analysis

n/a	Involved in the study
☐	☐ Functional and/or effective connectivity
☐	☐ Graph analysis
☐	☐ Multivariate modeling or predictive analysis

Functional and/or effective connectivity

Graph analysis

Multivariate modeling and predictive analysis

This checklist template is licensed under a Creative Commons Attribution 4.0 International License, which permits use, sharing, adaptation, distribution and reproduction in any medium or format, as long as you give appropriate credit to the original author(s) and the source, provide a link to the Creative Commons license, and indicate if changes were made. The images or other third party material in this article are included in the article's Creative Commons license, unless indicated otherwise in a credit line to the material. If material is not included in the article's Creative Commons license and your intended use is not permitted by statutory regulation or exceeds the permitted use, you will need to obtain permission directly from the copyright holder. To view a copy of this license, visit http://creativecommons.org/licenses/by/4.0/
